# HIG-Syn: a hypergraph and interaction-aware multigranularity network for predicting synergistic drug combinations

**DOI:** 10.1093/bioinformatics/btaf215

**Published:** 2025-07-15

**Authors:** Yuexi Gu, Jian Zu, Yongheng Sun, Louxin Zhang

**Affiliations:** School of Mathematics and Statistics, Xi’an Jiaotong University, Xi'an, Shaanxi 710049, People's Republic of China; School of Mathematics and Statistics, Xi’an Jiaotong University, Xi'an, Shaanxi 710049, People's Republic of China; School of Mathematics and Statistics, Xi’an Jiaotong University, Xi'an, Shaanxi 710049, People's Republic of China; Department of Mathematics and Centre for Data Science and Machine Learning, National University of Singapore, Singapore 119076, Singapore

## Abstract

**Motivation:**

Drug combinations can not only enhance drug efficacy but also effectively reduce toxic side effects and mitigate drug resistance. With the advancement of drug combination screening technologies, large amounts of data have been generated. The availability of large data enables researchers to develop deep learning methods for predicting drug targets for synergistic combination. However, these methods still lack sufficient accuracy for practical use, and most overlook the biological significance of their models.

**Results:**

We propose the HIG-Syn (hypergraph and interaction-aware multigranularity network for drug synergy prediction) model, which integrates a coarse-granularity module and a fine-granularity module to predict drug combination synergy. The former utilizes a hypergraph to capture global features, while the latter employs interaction-aware attention to simulate biological processes by modeling substructure–substructure and substructure–cell line interactions. HIG-Syn outperforms state-of-the-art machine learning models on our validation datasets extracted from the DrugComb and GDSC2 databases. Furthermore, the fact that five of the 12 novel synergistic drug combinations predicted by HIG-Syn are strongly supported by experimental evidence in the literature underscores its practical potential.

**Availability and implementation:**

The source code is available at https://github.com/gracygyx/HIGSyn

## 1 Introduction

Combination of drugs leads to improved therapeutic efficacy when their combined effect exceeds the additive effects of individual drugs in reducing toxicity and mitigating drug resistance. This phenomenon is known as drug synergy. Targeting multiple interacting pathways or biological processes (Szumilak *et al.* 2021), synergistic drug combinations are particularly effective in treating non-Mendelian diseases, such as cancer (Jaaks *et al.* 2022) and HIV (Tan *et al.* 2012).

High-throughput screening technologies have significantly advanced drug combination discovery, generating extensive datasets that include drug responses and synergy classifications. Key resources include the DCDB database (Liu *et al.* 2014), the first dedicated to multicomponent drugs, as well as other notable databases such as NCI-ALMANAC (a large matrix of antineoplastic agent combinations) (Holbeck *et al.* 2017), DrugComb (Zheng *et al.* 2021), and the recently published GDSC2 (Bashi *et al.* 2024). Complementary resources like DrugBank (Knox *et al.* 2024) and PubChem (Wang *et al.* 2009) provide comprehensive chemical and pharmacological data. Furthermore, multiomics datasets from NCI-60 and CCLE cell lines, available through CellMiner (Shankavaram *et al.* 2009), facilitate in-depth analyses of drug combinations. Collectively, these databases form a solid foundation for advancing the field of drug combination research.

Classical machine learning (ML) techniques are favored for their reliability and interpretability in drug combination prediction (Celebi *et al.* 2019, Lin *et al.* 2022). However, deep learning (DL) methods have significantly enhanced predictive capabilities by integrating advanced architectures (Chen and Zhang 2022). Feedforward neural networks are particularly effective in capturing complex nonlinear relationships in drug synergy prediction (Preuer *et al.* 2018, Kuru *et al.* 2022). Graph neural network-based models utilize graph representations to model drug molecules and their interactions (Wang *et al.* 2022, Chen and Zhang 2024). Hypergraph-based approaches excel in capturing multiway interactions (Liu *et al.* 2022).

Despite significant progress in drug combination prediction, several challenges remain. For example, models that rely exclusively on molecular fingerprints to encode drugs may miss essential structural information critical to understanding their anticancer efficacy (Yu *et al.* 2016). Even when molecular structure is incorporated, many models capture drug features from a global perspective, failing to identify key substructures that vary across different scales. Furthermore, most models treat drugs and cell lines independently, overlooking their interactions and the impact these interactions have on feature representation, which limits their biological relevance.

To address these challenges, we propose HIG-Syn (hypergraph and interaction-aware multigranularity network for drug synergy prediction), a framework that combines a coarse-grained hypergraph module for global triple interactions and a fine-grained interaction-aware attention module to model biological processes. This dual-module architecture effectively captures complex relationships, adaptively identifies biologically significant substructures across multiple scales, and models both global and pairwise interactions. HIG-Syn outperforms state-of-the-art models on our validation datasets extracted from the DrugComb and GDSC2 databases, with visualizations confirming its interpretability in identifying key anticancer substructures. Moreover, five of the 12 predicted drug combinations were experimentally validated, demonstrating its potential to advance drug discovery and precision medicine.

## 2 Materials and methods

### 2.1 Datasets

#### 2.1.1 Drug combination datasets

The drug synergy prediction task uses two large public datasets: DrugComb (accessed October 2024) and GDSC2 (accessed December 2024). The DrugComb dataset (available at https://drugcomb.org/) includes data from various screening studies, such as NCI-ALMANAC (Holbeck *et al.* 2017), O’Neil (O’Neil *et al.* 2016), FORCINA (Forcina *et al.* 2017), and CLOUD datasets (Licciardello *et al.* 2017). It comprises 8397 drugs, 2320 cell lines, and 33 tissue types, yielding 739 964 drug combinations.

The GDSC2 dataset (available at https://gdsc-combinations.depmap.sanger.ac.uk/) is the largest resource for cancer drug combination screening to date (Bashi *et al.* 2024). It comprises 109 drug combinations from AstraZeneca’s oncology portfolio, tested across 755 pan-cancer cell lines from 41 cancer types, with 68 816 distinct drug–drug–cell line combinations.

#### 2.1.2 Drug SMILES information

The SMILES representations for the drugs in the DrugComb and GDSC2 datasets were retrieved from the PubChem database (accessed October 2024, available at https://pubchem.ncbi.nlm.nih.gov/) using the pubchempy library (Version 1.0.4) in Python.

#### 2.1.3 Cell line profiles

Gene expression data (${\;\text{log}\;}_{2}(\text{RPKM}+1)$) for the cell lines were retrieved from the CellMinerCBD portal (Luna *et al.* 2021) (accessed October 2024, available at https://discover.nci.nih.gov/rsconnect/cellminercdb/).

To reduce dimensionality, we selected crucial genes from the LINCS L1000 project (accessed October 2024, available at https://lincsproject.org/LINCS/), which offers a “Landmark Gene Set” of 978 genes.

#### 2.1.4 Data preprocessing

Drug combination data: Drug synergy prediction is framed as a binary classification task, with synergistic combinations labeled as positive and nonsynergistic as negative. The DrugComb database provides classification results from four reference models: Bliss independence (Bliss 1939), Loewe additivity (Loewe 1953), HSA (Tang *et al.* 2015), and ZIP (Yadav *et al.* 2015). The GDSC2 database provides synergy scores based on HSA and Bliss independence models. A zero threshold is applied to convert scores into binary labels, with values above zero indicating synergy. The final label is determined by majority voting across the different reference models. To mitigate input order bias, both datasets are expanded to include (Drug 1, Drug 2, cell line) and (Drug 2, Drug 1, cell line) pairs. For duplicates, labels are determined by majority voting.

Drug data: The drug’s SMILES string was converted into a 1024-bit ECFP6 fingerprint (radius 6) using the RDKit package (Version 2023.3.2) in Python, providing a fixed-length descriptor of the drug’s structural features, which was then utilized to calculate the reconstruction loss.

The molecular structure is represented as a graph, where atoms are nodes and bonds are edges. Each atom is described by a 78-dimensional feature vector that encodes properties such as atom type, degree, hydrogen count, implicit valence, and aromaticity, as described by Wang *et al.* (2022). A summary of these features is provided in Table 1, and they are used as input for the model.

**Table 1.** Atomic features of the drug graph.

Feature	Dimension	Description
Atom type	43	One-hot encoding of atom types (e.g. C, N, O, etc.)
Degree	11	One-hot encoding of atom degree (0–10)
Number of hydrogens	11	One-hot encoding of the number of hydrogens (0–10)
Implicit valence	11	One-hot encoding of implicit valence (0–10)
Aromaticity	1	Whether the atom is in an aromatic system (binary: 0 or 1)

Cell line data: The genes are selected based on the Landmark Gene Set, and the gene expression data is subsequently preprocessed using z-score normalization.

### 2.2 Framework of HIG-Syn

#### 2.2.1 Overview

**Figure 1.**
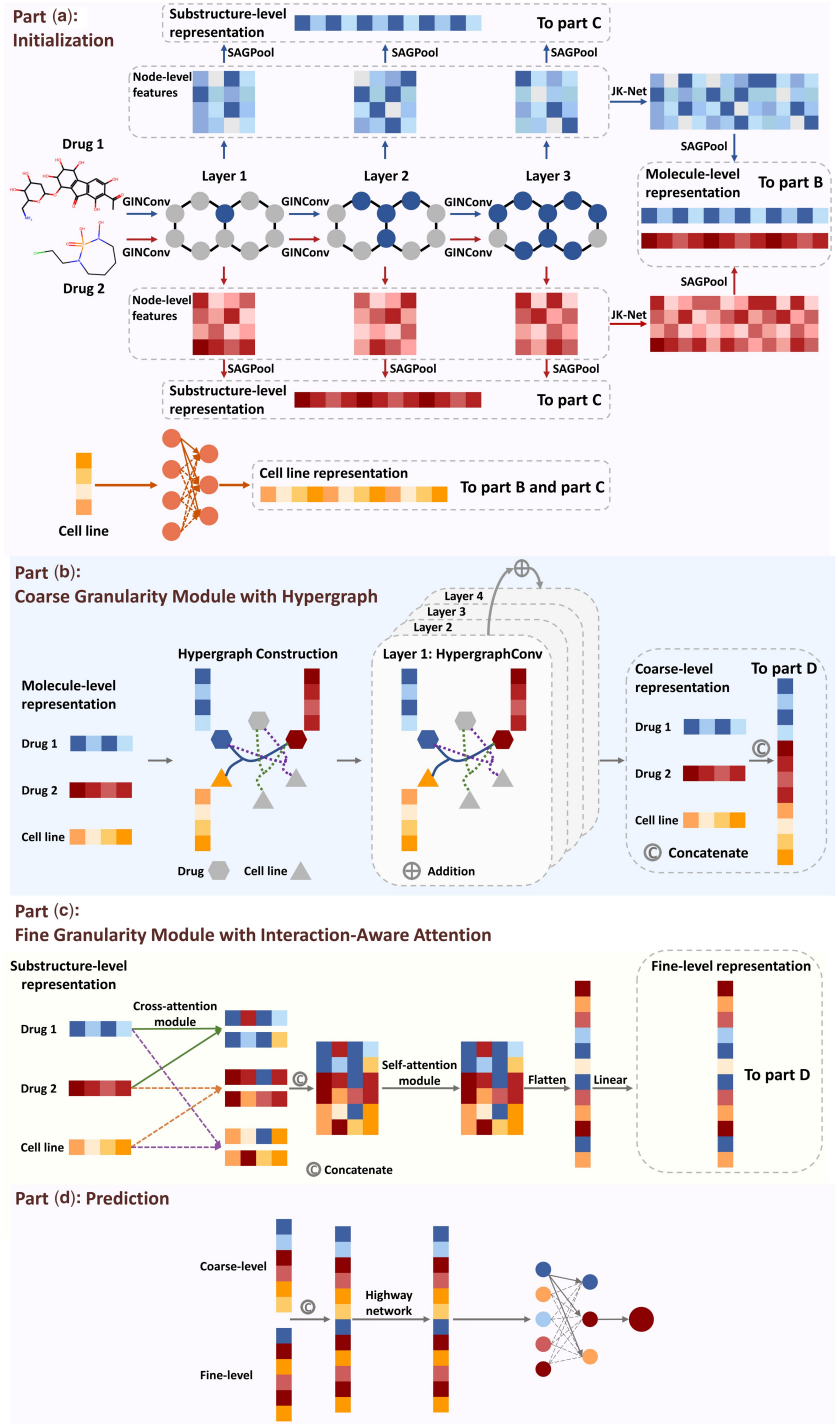
The architecture of HIG-Syn. The model input consists of drug molecule graphs for two drugs and cell line gene expression data. (a) The initialization module extracts substructure- and molecular-level features for drugs using GIN layers with SAGPool, while MLP processes cell line features. (b) The coarse-granularity module employs a hypergraph for global features. (c) The fine granularity module focuses on substructure interactions using interaction-aware attention to local features. (d) In the prediction module, coarse and fine features are integrated via a highway network, with MLP layers predicting drug combination outcomes.

Figure 1 illustrates the HIG-Syn model. It has four key modules: (A) initialization, (B) coarse granularity module with hypergraph, (C) fine granularity module with interaction-aware attention, and (D) prediction. (Mathematical details of these modules are given in Section S1 of the Supplementary Material.)

**Initialization module**: Drugs are represented as 2D molecular graphs, where nodes correspond to atoms and edges represent bonds. The node features are encoded as a 78-dimensional vector to capture atomic properties (details in Table 1).

The node features are then updated by aggregating information from neighboring nodes in the Graph Isomorphism Network (GIN) layer. JK-Net (Xu *et al.* 2018) enables cross-layer connections to combine multiscale features. Self-Attention Graph Pooling (SAGPool) (Lee *et al.* 2019) uses a one-layer Graph Convolutional Network (GCN) to calculate node importance, extracting subgraph-level features that represent substructures embeddings and graph-level features that represent molecular-level embeddings.

Cell line embeddings are obtained using a two-layer MLP (Multilayer Perceptron) applied to gene expression data.

**Coarse granularity module with hypergraph**: In the coarse-granularity module, molecular-level drug embeddings and cell line embeddings are processed through a four-layer hypergraph, where nodes represent drugs and cell lines, and hyperedges capture synergistic drug–drug–cell line interactions. Residual connections in the final layer combine input features with the output of the third layer before convolution, resulting in a coarse-level global representation of drug combinations.

**Figure 2.**
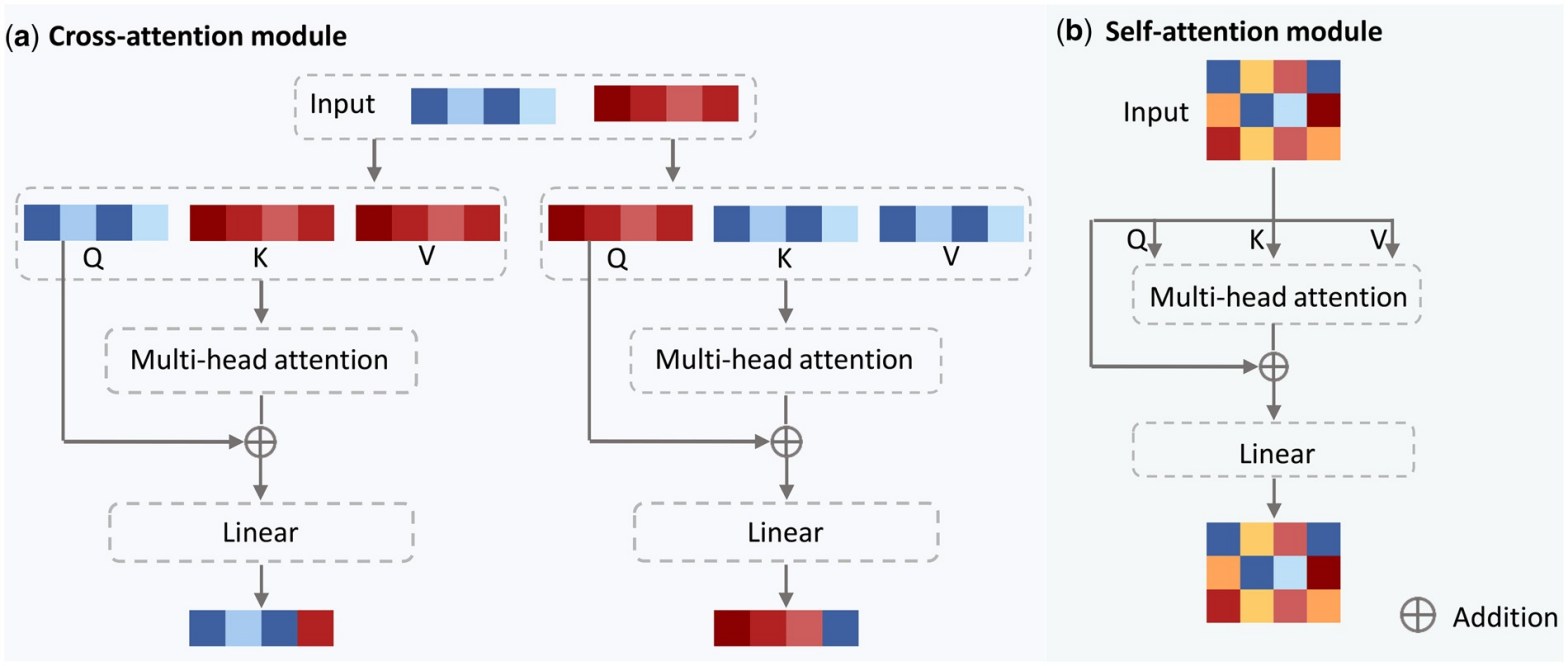
The interaction-aware attention module. (a) Cross-attention module for computing substructure–substructure and substructure–cell line interactions. (b) Self-attention module for integrating interaction-aware features and generating the final fine-level representations.

**Fine granularity module with interaction-aware attention**: The fine-granularity module captures interactions between drug substructures and cell lines through cross-attention and self-attention mechanisms (Fig. 2). Subgraph-level features are obtained by concatenating the outputs of each GIN layer from the initialization module. Cross-attention (Fig. 2a) models interactions between drug substructures and substructure–cell line pairs using four-head attention. The resulting six vectors are then processed via self-attention (Fig. 2b) and a shared-weight linear layer, where each vector shares the same set of weights, to produce the final fine-level representations.

**Prediction module**: The prediction module consists of a highway network (Srivastava *et al.* 2015) and an MLP. The input is a concatenated vector of coarse-level and fine-level representations, which are adaptively fused in the highway network with transformation gates, which control the extent of transformation, and carry gates, which preserve essential information. A two-layer MLP then makes the prediction.

**Loss function**: The loss function is a weighted sum of classification, reconstruction, and contrastive losses (Section S1.7 of the Supplementary Material). Classification loss is computed using cross-entropy. Reconstruction loss measures the difference between the hypergraph-based and original similarity matrices, derived from cosine similarity of drug ECFP6 fingerprints and gene expression vectors. Contrastive loss separates positive and negative samples in the hypergraph embedding space, with an equal number of randomly selected negative samples.

### 2.3 Experimental settings

#### 2.3.1 Global settings

Hyperparameter tuning was performed using five-fold cross-validation on the DrugComb dataset. Key hyperparameters included the hidden units in the three GIN layers ([128, 128, 128]), the two MLP layers for cell line feature extraction ([512, 384]), and the hypergraph coarse module architecture ([768, 384, 384, 384]). Additional settings included the number of heads in the multi-head attention (4), a dropout rate of 0.3, and a learning rate of 0.0004. These optimal configurations were selected based on performance across various hyperparameter settings (Supplementary Table S1 and Supplementary Fig. S1). The model was trained using the Adam optimizer for up to 2000 epochs with early stopping to mitigate overfitting. All experiments were implemented in PyTorch (Version 2.4.0).

#### 2.3.2 Evaluation metrics

Eight classification metrics were used for the evaluation: AUC-ROC (Area Under the Curve-Receiver Operating Characteristic Curve), AUC-PR (Area Under the Precision-Recall Curve), accuracy (ACC), balanced accuracy (BACC), F1 score, precision, recall, and Cohen’s kappa (kappa).

#### 2.3.3 Cross-validation design

We evaluated the performance of our model using a five-fold cross-validation, with data divided into training, validation, and test sets in a 3:1:1 ratio. The model is evaluated in three scenarios and compared with 11 benchmark models (Section S2 of the Supplementary Material) on the DrugComb and GDSC2 datasets.

- Random cross-validation scenario: We randomly divided all drug–drug–cell line triplets into five subsets. Here, drug combinations (Drug 1, Drug 2, Cell line) in the test set did not overlap with those in the training set.
- Leave-drug-pair-out scenario: To ensure that drug pairs (Drug 1, Drug 2) in the test set were excluded from the training set, we enumerated all drug pairs and assigned them to five mutually exclusive folds. This way, each fold contained only the samples involving the assigned drug pairs.
- Leave-cell-line-out scenario: All cell lines were divided into five folds and the samples involving assigned cell lines were uniquely assigned to the corresponding fold to ensure that the model was tested on entirely unseen cell lines.

#### 2.3.4 Ablation study

We conducted 14 sets of ablation experiments (Section S3 of the Supplementary Material) to evaluate the contributions of various components to model performance from the following three primary perspectives.

- The impact of the core components of the model, including the coarse-granularity module, the residual connection in the coarse-granularity module, the fine-granularity module, and the highway network for adaptive fusion.
- The impact of the reconstruction term and the contrastive term in the loss function, as well as their combined effect.
- The influence of modifications to the GIN module, including the substitution of GIN with Graph Attention Networks (GAT) or GCN, and the replacement of the graph-level aggregator from SAGPool to AddPool, MaxPool, and MeanPool.

## 3 Results

### 3.1 Comparison with existing models

To evaluate the classification performance of our model, we compared it with 11 comparative models (four classical ML and seven advanced DL models) (Section S2 of the Supplementary Material) using two datasets: DrugComb and GDSC2. Performance was assessed in two types of scenarios: random CV scenario with five-fold cross-validation and more challenging leave-out scenarios, including leave-drug-pair-out and leave-cell-line-out.

The mean and variance of the eight classification metrics for our model and the comparison models in the random CV scenario on the DrugComb and GDSC2 datasets are shown in Table 2 and Supplementary Table S2, respectively. Below is a summary of the classification performance on both datasets.

**Table 2.** Performance comparison of HIG-Syn and 11 baseline models on the DrugComb dataset.

Model	AUC-ROC	AUC-PR	ACC	BACC	F1	Precision	Recall	Kappa
HIG-Syn	**0.954** ± 0.003	**0.923** ± 0.005	**0.912** ± 0.004	**0.898** ± 0.005	**0.860** ± 0.006	**0.860** ± 0.006	**0.861** ± 0.009	**0.796** ± 0.009
EN	0.672±0.001	0.494±0.002	0.703±0.001	0.550±0.002	0.223±0.007	0.637±0.007	0.135±0.005	0.127±0.004
GBM	0.677±0.001	0.509±0.003	0.707±0.001	0.552±0.001	0.224±0.003	0.678±0.006	0.134±0.002	0.134±0.003
RF	0.744±0.001*	0.582±0.001	0.740±0.0004*	0.641±0.0004*	0.476±0.001*	0.651±0.003	0.375±0.001*	0.319±0.001*
XGBoost	0.739±0.001	0.596±0.002*	0.738±0.001	0.621±0.001	0.424±0.002	0.689±0.004*	0.306±0.002	0.286±0.002
DeepDDS-GAT	0.773±0.004	0.641±0.005	0.751±0.003	0.671±0.010	0.534±0.021	0.653±0.023	0.454±0.040	0.372±0.012
DeepDDS-GCN	0.759±0.102	0.631±0.126	0.756±0.038	0.645±0.076	0.442±0.221	0.742 ± 0.010	0.346±0.180	0.329±0.169
HypergraphSynergy	0.773±0.003	0.652±0.005	0.714±0.004	0.703 ± 0.003	0.597 ± 0.004	0.536±0.005	0.672 ± 0.008	0.379±0.006
MatchMaker	0.795 ± 0.003	0.674 ± 0.004	0.769 ± 0.002	0.688±0.007	0.561±0.013	0.700±0.010	0.469±0.022	0.413 ± 0.010
MFSynDCP	0.608±0.006	0.420±0.006	0.690±0.002	0.511±0.002	0.054±0.012	0.709±0.031	0.028±0.006	0.031±0.006
MLP	0.726±0.005	0.579±0.006	0.709±0.008	0.662±0.003	0.537±0.005	0.539±0.017	0.536±0.020	0.325±0.009
MPFFPSDC	0.710±0.006	0.551±0.007	0.716±0.003	0.634±0.008	0.476±0.020	0.569±0.014	0.411±0.036	0.289±0.011
SDDSynergy	0.723±0.014	0.567±0.021	0.728±0.008	0.616±0.017	0.418±0.040	0.642±0.016	0.311±0.045	0.268±0.033

The best traditional machine learning and deep learning methods are marked with an asterisk (*) and underlined, respectively. Bold values are the performance of HIG-Syn.

For the DrugComb dataset (Table 2), Random Forest (RF) performs best among ML models, excelling in all metrics except AUC-PR and precision. XGBoost outperforms in AUC-PR and precision. Among DL methods, MatchMaker optimizes AUC-ROC, AUC-PR, ACC, and kappa, while HypergraphSynergy excels in BACC, F1, and recall. Our model outperforms all 11 comparison models across every metric, improving the best-performing models by 20% in AUC-ROC, 38% in AUC-PR, 15% in ACC, 28% in BACC, 44% in F1, 16% in precision, 28% in recall, and 93% in kappa.

For the GDSC2 dataset (Supplementary Table S2), RF outperforms other ML models across all metrics. DeepDDS-GCN, the leading DL model, exceeds ML models in all metrics except recall. Our model outperforms all comparisons, achieving a 2% improvement in AUC-ROC and AUC-PR, a 4% improvement in ACC, BACC, F1, precision, and recall, and a 9% improvement in kappa.

### 3.2 Performance comparison in leave-out scenarios

We also evaluated our model in the more challenging leave-out scenarios. For the DrugComb dataset, the results are given in Table 3. Our model achieves optimal performance in all metrics under the leave-drug-pair-out scenario. In the leave-cell-line-out scenario, it ranks first in BACC and F1, second in AUC-ROC and recall, and third in AUC-PR.

**Table 3.** Model performance in the leave-out scenario on the DrugComb dataset.

Model	Leave-drug-pair-out					Leave-cell-line-out				
	AUC-ROC	AUC-PR	BACC	F1	Recall	AUC-ROC	AUC-PR	BACC	F1	Recall
HIG-Syn	**0.787** ± 0.003	**0.661** ± 0.010	**0.711** ± 0.003	**0.607** ± 0.005	**0.699** ± 0.016	**0.694** ± 0.009	**0.518** ± 0.029	**0.642** ± 0.016	**0.539** ± 0.012	**0.706** ± 0.045
EN	0.667± 0.009	0.488± 0.010	0.548± 0.003	0.220± 0.009	0.133± 0.006	0.635± 0.015	0.431± 0.015	0.548± 0.012	0.265± 0.050	0.186± 0.052
GBM	0.670± 0.008	0.495± 0.009	0.547± 0.002	0.208± 0.006	0.124± 0.005	0.641± 0.011	0.452± 0.010	0.553± 0.003	0.261± 0.025	0.173± 0.026
RF	0.724± 0.005*	0.560± 0.008*	0.622± 0.002*	0.438± 0.005*	0.337± 0.006*	0.701± 0.008*	0.546± 0.009*	0.622± 0.004*	0.446± 0.012*	0.357± 0.020*
XGBoost	0.708± 0.008	0.545± 0.011	0.602± 0.004	0.386± 0.011	0.278± 0.010	0.661± 0.008	0.487± 0.018	0.593± 0.006	0.395± 0.026	0.318± 0.048
DeepDDS-GAT	0.732± 0.006	0.582± 0.011	0.643± 0.008	0.486± 0.015	0.407± 0.027	0.678± 0.011	0.504± 0.013	0.614± 0.007	0.454± 0.017	0.412± 0.035
DeepDDS-GCN	0.781± 0.008	0.653± 0.013	0.672± 0.011	0.531± 0.023	0.429± 0.031	0.694 ± 0.008	0.529 ± 0.006	0.613± 0.006	0.428± 0.021	0.342± 0.031
HypergraphSynergy	0.731± 0.004	0.593± 0.012	0.673± 0.005	0.564 ± 0.007	0.662 ± 0.014	0.617± 0.009	0.432± 0.015	0.557± 0.036	0.487 ± 0.012	0.800 ± 0.122
MatchMaker	0.784 ± 0.005	0.659 ± 0.010	0.677 ± 0.007	0.541± 0.014	0.442± 0.020	0.686± 0.007	0.517± 0.025	0.614 ± 0.012	0.438± 0.021	0.364± 0.024
MFSynDCP	0.612± 0.015	0.423± 0.012	0.516± 0.006	0.083± 0.038	0.046± 0.022	0.591± 0.011	0.384± 0.012	0.506± 0.007	0.062± 0.068	0.037± 0.043
MLP	0.686± 0.006	0.528± 0.014	0.634± 0.007	0.503± 0.013	0.526± 0.037	0.622± 0.009	0.444± 0.017	0.594± 0.004	0.451± 0.009	0.471± 0.036
MPFFPSDC	0.677± 0.011	0.507± 0.009	0.601± 0.013	0.407± 0.034	0.328± 0.045	0.630± 0.016	0.428± 0.018	0.562± 0.022	0.328± 0.074	0.259± 0.077
SDDSynergy	0.694± 0.020	0.527± 0.024	0.608± 0.019	0.414± 0.046	0.325± 0.055	0.641± 0.011	0.446± 0.019	0.562± 0.012	0.304± 0.056	0.219± 0.057

The best traditional machine learning and deep learning methods are marked with an asterisk (*) and underlined, respectively. The bold values are the performance of HIG-Syn.

For the GDSC2 dataset (Supplementary Table S3), in the leave-drug-pair-out scenario, our model ranks second in all metrics except BACC, where it ranks seventh, 4% lower than the top model. In the leave-cell-line-out scenario, it is optimal for AUC-PR, recall, and F1, second for AUC-ROC, and fourth for BACC, 1% below the best-performing model.

### 3.3 Ablation analysis

We conducted ablation experiments to evaluate the impact of model components and model variants on classification performance.

#### 3.3.1 Impact of module removal on performance

**Figure 3.**
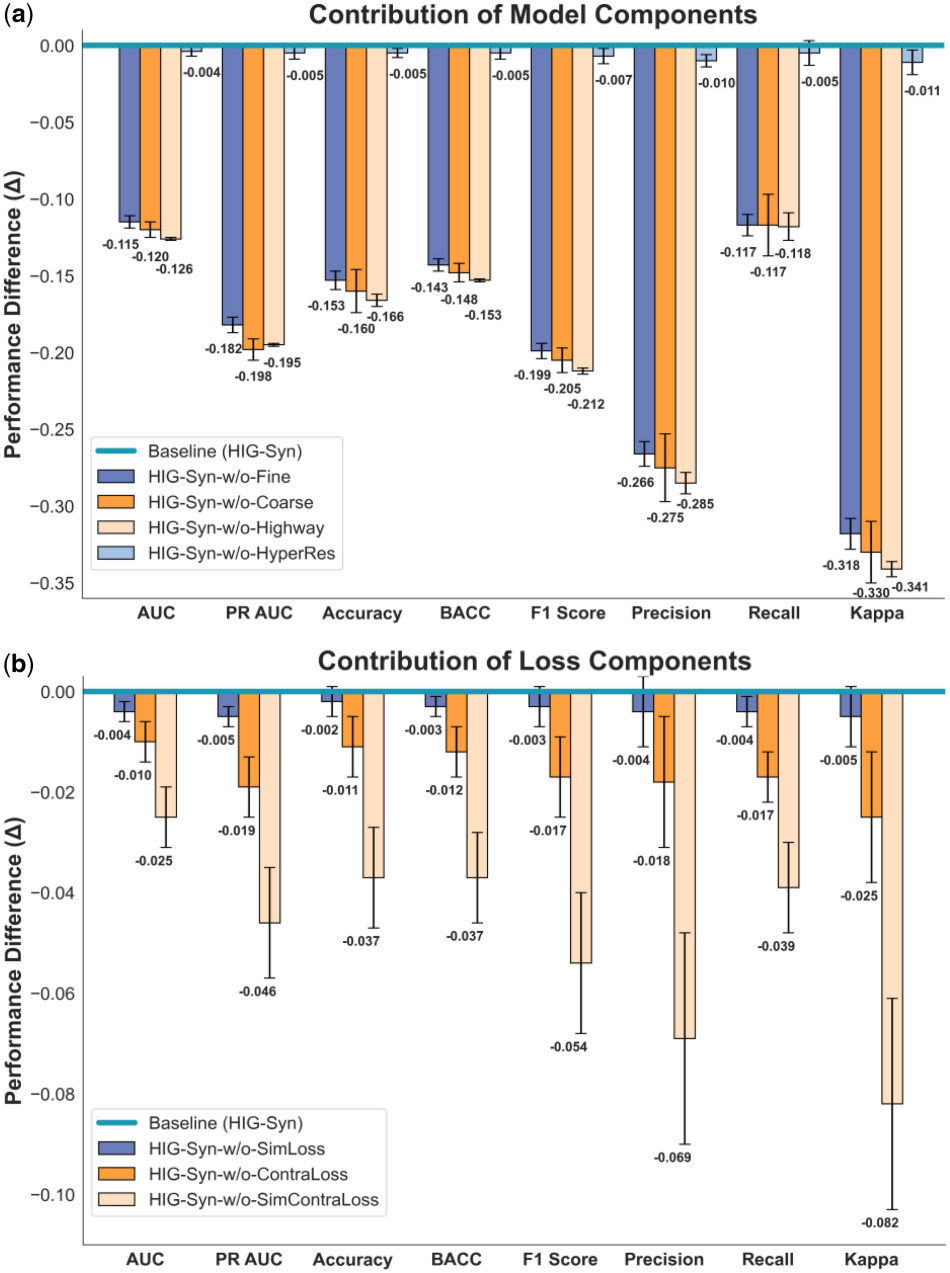
Impact of component removal on model performance. (a) Impact of module removal on model performance across classification metrics. (b) Effect of removing components from the loss function on model performance.

The importance of each module and each loss function was assessed by measuring performance changes when each module was removed. Figure 3 shows the impact of the performance on classification metrics, with longer downward bars indicating a greater decline, highlighting the importance of the removed module.

Figure 3a illustrates the impact of the coarse granularity, fine granularity, and highway network, as well as residual connections in the final hypergraph layer. The highway network has the greatest impact, followed by the coarse-granularity module. Residual connections have minimal effect. Kappa is the most sensitive metric, while AUC and recall remain stable.

The model’s loss function includes classification, reconstruction, and contrastive losses. Figure 3b shows that combining reconstruction and contrastive losses results in a greater performance boost than using either loss alone. Additionally, Fig. 3a highlights that omitting a loss component affects performance more than removing a model module.

#### 3.3.2 Impact of model variants on performance

We evaluated the impact of replacing GIN with GCN, GAT with different heads (1, 2, and 4 heads), and different aggregators (add, max, and mean pooling). Table 4 shows that GCN outperforms GAT, but GIN is the best. Mean pooling outperforms add and max pooling, though it still falls short of SAGPool in the original model.

**Table 4.** Impact of model variants on DrugComb dataset.

Model	AUC-ROC	AUC-PR	ACC	BACC	F1	Precision	Recall	Kappa
HIG-Syn	**0.954** ± 0.003	**0.923** ± 0.005	**0.912** ± 0.004	**0.898** ± 0.005	**0.860** ± 0.006	**0.860** ± 0.006	**0.861** ± 0.009	**0.796** ± 0.009
HIG-Syn-GCN	0.938±0.004	0.897±0.008	0.892±0.007	0.876±0.008	0.829±0.012	0.825±0.015	0.833±0.013	0.750±0.017
HIG-Syn-GAT-1	0.945±0.003	0.909±0.005	0.901±0.005	0.886±0.005	0.843±0.007	0.841±0.008	0.846±0.007	0.771±0.010
HIG-Syn-GAT-2	0.947±0.004	0.914±0.007	0.905±0.006	0.891±0.006	0.850±0.009	0.846±0.014	0.853±0.007	0.780±0.014
HIG-Syn-GAT-4	0.946±0.004	0.910±0.006	0.901±0.006	0.886±0.006	0.843±0.008	0.838±0.012	0.848±0.009	0.770±0.012
HIG-Syn-AddPool	0.843±0.004	0.745±0.005	0.774±0.006	0.759±0.004	0.667±0.004	0.624±0.011	0.716±0.010	0.497±0.010
HIG-Syn-MaxPool	0.869±0.014	0.783±0.019	0.805±0.016	0.789±0.015	0.707±0.020	0.671±0.027	0.747±0.016	0.561±0.033
HIG-Syn-MeanPool	0.951±0.002	0.919±0.001	0.909±0.001	0.895±0.002	0.856±0.002	0.854±0.008	0.857±0.007	0.789±0.003

The best results are highlighted in bold.

### 3.4 Order independence

**Figure 4.**
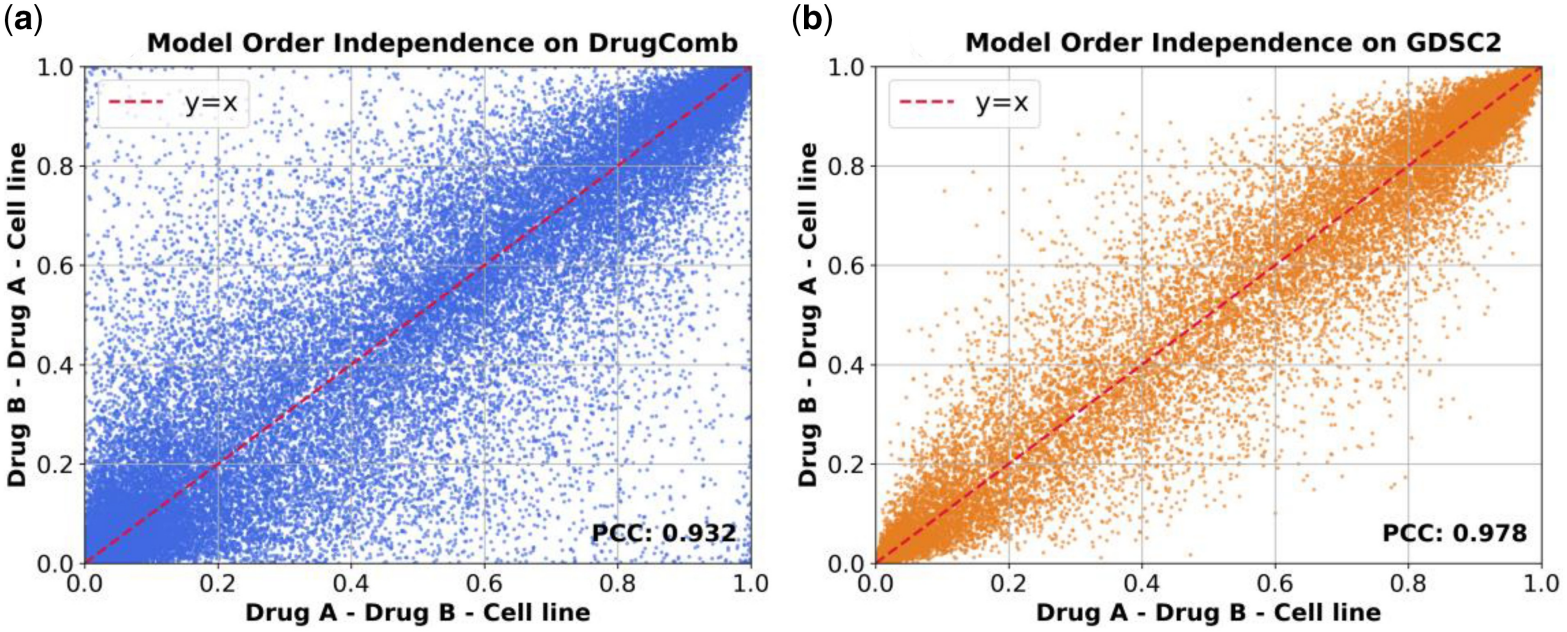
Scatter plot of predicted probabilities under different drug input orders: (a) the DrugComb dataset and (b) the GDSC2 dataset.

We tested the impact of input order on the model by calculating the Pearson correlation coefficients (PCC) for inputs (Drug 1, Drug 2, cell line) and (Drug 2, Drug 1, cell line) (Fig. 4). The model showed a PCC of 0.932 for the DrugComb dataset and 0.978 for the GDSC2 dataset, demonstrating that the model is insensitive to the order of input.

### 3.5 Visualization of important substructures

**Figure 5.**
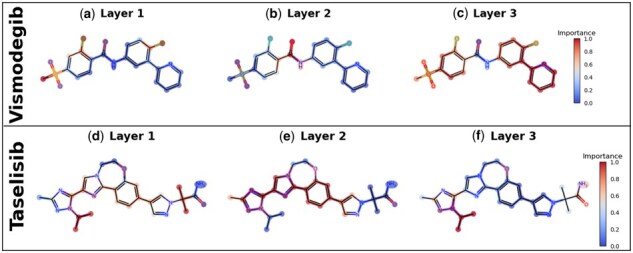
Key molecular structures identified by GIN. (a–c) Substructure importance of Vismodegib across GIN layers. (d–f) Substructure importance of Taselisib across GIN layers.

In our model, drug features at each GIN layer are extracted using SAGPooling, which calculates atom-level importance via the GCN layer. We selected Vismodegib from DrugComb and Taselisib from GDSC2 as representative cancer drugs. In Fig. 5, the red regions highlight the key substructures identified at each GIN layer.

The key substructures of Vismodegib include the amide moiety in Fig. 5b and the pyridine ring, benzene ring, and azepine ring in Fig. 5c, all extensively studied for their anticancer properties (Singh *et al.* 2016). Pyridine is used in anticancer agents by inhibiting antiapoptotic proteins, stimulating apoptotic proteins, causing DNA damage, and generating reactive oxygen species to induce tumor cell death (Elagamy *et al.* 2023).

Taselisib’s key substructures include alkyl groups in Fig. 5d, dimethylamino groups, azepines, aromatic rings, and pyrazoline in Fig. 5f. These substructures have been extensively validated in numerous studies for their anticancer activity (Sadashiva *et al.* 2012). For example, pyrazoline, a widely used scaffold in novel anticancer agents, holds promise for minimizing side effects and overcoming drug resistance when hybridized with various heterocycles (Haider *et al.* 2022).

### 3.6 Visualization of embedding vectors from different modules

**Figure 6.**
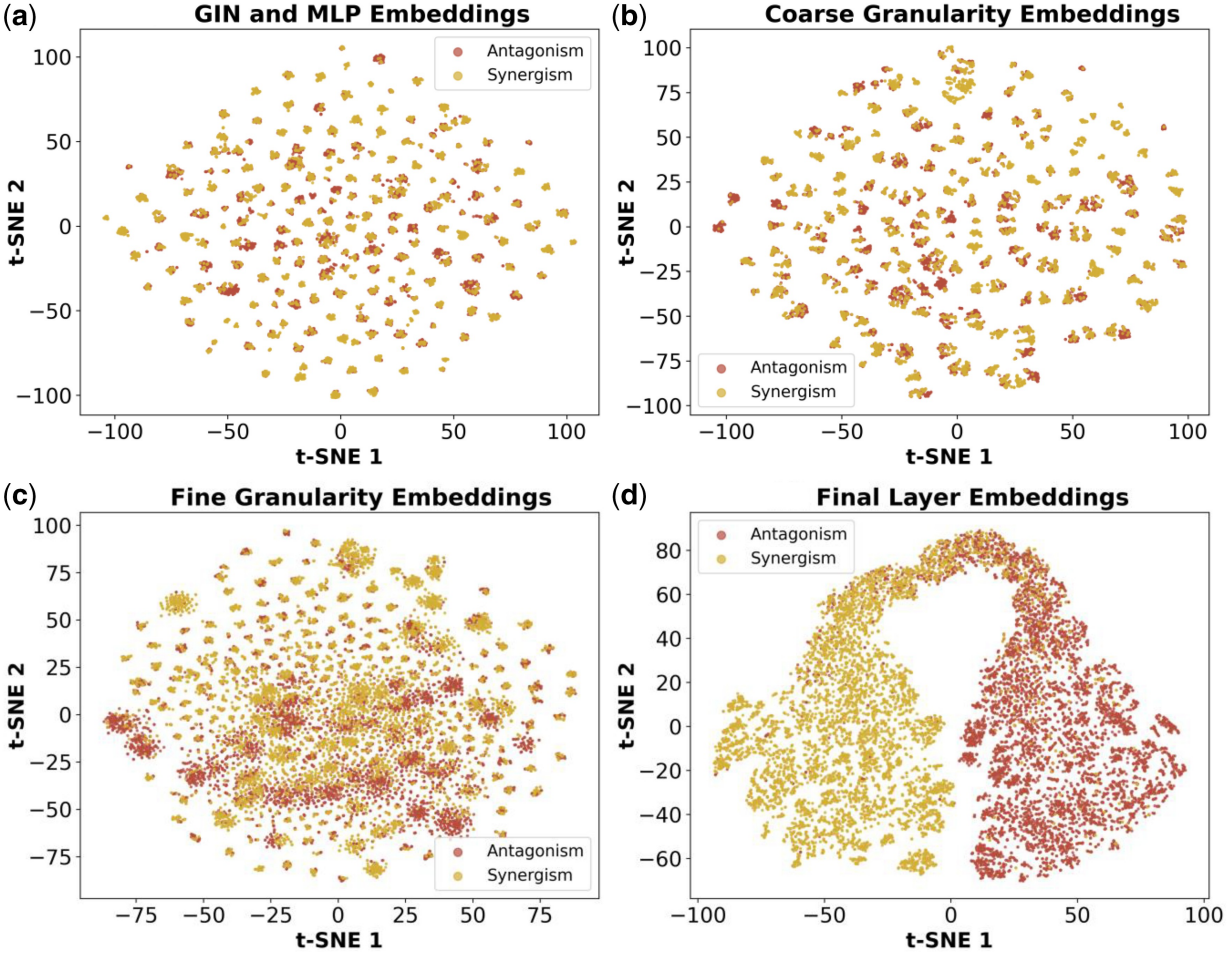
T-SNE visualization of drug–drug–cell line triplet representations across different HIG-Syn modules on GDSC2 dataset. (a) Initialization module output. (b) Coarse-granularity module output with hypergraph. (c) Fine-granularity module output with interaction-aware attention. (d) Final representation vectors for prediction.

The output of the initialization module, the coarse-granularity module, the fine-granularity module, and the final layer were visualized in Fig. 6 (DGSC2 dataset) and Supplementary Fig. S2 (DrugComb dataset).

In the Initialization and coarse-granularity modules (Fig. 6a and b), the separation between sample types is less distinct due to the shared module for drug representations. In contrast, the fine-granularity module (Fig. 6c), which incorporates drug–drug and drug–cell line interactions, achieves a clearer distinction. In the final layer (Fig. 6d), the model demonstrates significantly improved classification performance, with a pronounced separation of embedding vectors between the two classes.

### 3.7 Discovering novel drug combinations

For the DrugComb and GDSC2 datasets, we generated novel drug–drug–cell line combinations by pairing the five most common drugs with all others in the three largest cell lines for each dataset. Using a model trained on the full dataset, we predicted the synergistic probabilities for novel combinations. The selected cell lines for DrugComb were HT-29, SK-OV-3, and T-47D, and for GDSC2 were C32, MHH-ES-1, and HT-29. Table 5 presents the literature validation of the top six predicted synergistic combinations per dataset, while Supplementary Table S4 summarizes the predicted probabilities from baselines.

**Table 5.** Predicted outcomes from the 11 compared models on the 12 novel combinations.

Dataset	Drug pair	Cell line	Reference
DrugComb	Vincristine + Crizotinib	HT-29	Sampson *et al.* (2022) (*)
	Carboplatin + Ruxolitinib	SK-OV-3	Han *et al.* (2018) (***)
	Crizotinib + Erlotinib	SK-OV-3	Van der Steen *et al.* (2019) (*)
	Navitoclax + Ruxolitinib	SK-OV-3	Pemmaraju *et al.* (2025) (*)
	Dactolisib + Crizotinib	T-47D	Singh *et al.* (2022) (*)
	1,25-dihydroxy vitamin D3 + Ruxolitinib	T-47D	Lim *et al.* (2018) (**)
GDSC2	AZD6738 + Paclitaxel	C32	Kim *et al.* (2021) (**)
	AZD1775 + Doxorubicin	HT-29	Hirai *et al.* (2010) (**)
	AZD6738 + Etoposide	HT-29	Devin *et al.* (2020) (*)
	5-Fluorouracil + AZD1775	HT-29	Webster *et al.* (2017) (***)
	AZD4320 + AZD5991	MHH-ES-1	Menendez *et al.* (2022) (*)
	5-Fluorouracil + AZD6738	MHH-ES-1	Wichert *et al.* (2024) (*)

The symbols *, **, and *** indicate that the literature validates the synergistic properties of the listed drug pair in (i) a cell line unrelated to the listed tissue or disease, (ii) the tissue or disease associated with the listed cell line, and (iii) the listed cell line, respectively.

HT29, a colon adenocarcinoma cell line with a P53 mutation, has been used to study the synergistic effects of AZD1775, a WEE1 inhibitor, combined with chemotherapeutic agents. One study (Webster *et al.* 2017) showed that AZD1775, when combined with 5-fluorouracil (5-FU), increased γH2AX (a marker of double-strand DNA breakage) expression and enhanced caspase-3-dependent apoptosis, amplifying 5-FU’s cytotoxic effects. Similarly, AZD1775 synergized with Doxorubicin, a topoisomerase II inhibitor, in P53-mutated colon cancer cell lines, enhancing its cytotoxicity and efficacy (Hirai *et al.* 2010).

SK-OV-3, a serous ovarian cancer cell line, has been used to study the effects of Ruxolitinib, a JAK1/2 inhibitor, in combination with chemotherapeutic agents. A study (Han *et al.* 2018) demonstrated that Ruxolitinib inhibits ovarian cancer cell viability and enhances Carboplatin’s antitumor activity. The combination significantly reduced tumor growth and exhibited synergistic effects in SK-OV-3 and other ovarian cancer cell lines.

T-47D, a breast cancer cell line, relies on ER and HER signaling for growth (Kirkegaard *et al.* 2014). Ruxolitinib, a JAK1/2 inhibitor, and Calcitriol (1,25-dihydroxy vitamin D3), the active form of vitamin D, have synergistic effects in breast cancer. A study (Lim *et al.* 2018) demonstrated that their combination inhibits cell proliferation, regardless of ER status, through downregulation of JAK2, phosphorylated JAK2, c-Myc, and CCND1, as well as the induction of apoptosis regulators like Bcl-2, Bcl-2-like Protein 1, and Caspase-3.

C32 is a melanoma cell line, and Paclitaxel, a common chemotherapeutic, has been studied with Ceralasertib (AZD6738), an ATR kinase inhibitor. A clinical study (Kim *et al.* 2021) of 57 patients, including 33 with PD1/L1-resistant melanoma, showed that the combination of Ceralasertib and Paclitaxel exhibits antitumor activity and sustained efficacy in advanced melanoma.

Although the synergistic effects of the remaining seven drugs in the respective cell lines are unvalidated, their mechanisms have been studied in other tissues, supporting our predictions.

- The combination of Vincristine and Crizotinib inhibits tumor cell proliferation and blocks the RAS/MAPK, PI3K/AKT, and JAK/STAT3 signaling pathways in nonsmall cell lung cancer EML4-ALK V1 cells (Sampson *et al.* 2022).
- The combination of Crizotinib (c-Met pathway inhibitor) and Erlotinib (EGFR pathway inhibitor) shows synergistic effects in various tumor models. One study (Van der Steen *et al.* 2019) in nonsmall cell lung cancer demonstrated reduced phosphorylation of MAPK and PI3K/Akt/mTOR pathway targets, leading to strong inhibition and increased cell death. In glioblastoma (Goodwin *et al.* 2018), the combination inhibited tumor growth, phosphorylated EGFRvIII, Met, AKT, and MAPK, and suppressed neurosphere growth.
- Navitoclax induces apoptosis in malignant cells in myelofibrosis. Its synergistic effect with Ruxolitinib was demonstrated in a clinical trial involving MF patients (Pemmaraju *et al.* 2025).
- The synergistic effect of Dactolisib and Crizotinib in a triple-negative breast cancer model (Singh *et al.* 2022) reduced MAPK, PI3K/Akt, JAK/STAT pathway activity, and EMT markers.
- AZD6738, an ATR inhibitor, induces apoptosis, inhibits tumor cell proliferation, and causes double-strand breaks in cHL cell lines. Its combination with Etoposide shows synergistic effects in these cell lines (Devin *et al.* 2020).
- AZD4320, a dual BCL-2 and BCL-XL inhibitor, and AZD5991, which targets MCL-1, induce cell death in acute lymphoblastic leukemia cells when combined (Menendez *et al.* 2022).
- The synergy of AZD6738 and 5-FU in a p53-mutated colorectal cancer model is mediated by AZD6738’s inhibition of 5-FU-induced G2/M checkpoint activation, enhanced apoptosis, and increased DNA damage, reducing cell survival (Wichert *et al.* 2024).

Furthermore, we examined synergistic drug combinations predicted by RF, XGB, and MatchMaker—three top baseline models—but not by our model. Two such combinations were identified in DrugComb and nine in GDSC2 (Supplementary Table S5). A comprehensive literature review found no published evidence supporting the synergistic effects of these drug pairs in any specific cell line. These results further highlight the robustness of our model in reducing false positive predictions.

## 4 Discussion and conclusion

We propose the HIG-Syn model for predicting drug synergy in combination therapies. To capture synergy across multiple scales, the model integrates a coarse-granularity module based on hypergraphs and a fine-granularity module using interaction-aware attention. The fine-grained module captures drug–substructure and drug–cell line interactions, while the coarse module extracts global features.

By extracting features at multiple scales, HIG-Syn improves both classification accuracy and model stability. On the DrugComb and GDSC2 datasets, the model outperforms existing methods across all eight evaluation metrics. Visualization of substructure importance scores demonstrates the model’s ability to identify key anticancer substructures with biological relevance. Additionally, five out of 12 novel synergistic drug combinations were experimentally validated, highlighting the model’s practical potential.

In more challenging scenarios, such as the leave-out experiment, the model’s performance is suboptimal. This is likely due to isolated points in the hypergraph representation of drugs or cell lines not encountered during training. To address this, incorporating adversarial learning or adding pseudo-hyper edges could help mitigate this issue.

Overall, the model’s accuracy and ability to identify biologically significant features suggest strong potential for further exploration. It could be extended to predict drug toxicity, resistance, or interactions, offering valuable insights for more effective drug regimens.

In conclusion, HIG-Syn shows great promise as a tool for the prediction of drug synergy. Its potential to improve drug discovery, advance personalized medicine, and enhance our understanding of molecular interactions underscores its value. Continued refinement of this model could drive innovations in therapeutic strategies, facilitating the development of more effective and safer drug combinations for a wide range of diseases.

## Author contributions

Yuexi Gu (conceptualization, investigation, methodology, software, writing—original draft, writing—review and editing), Jian Zu (supervision, writing—review and editing, funding acquisition), Yongheng Sun (investigation, methodology, software), Louxin Zhang (supervision, conceptualization, writing—review and editing)

## Supplementary Material

btaf215_Supplementary_Data

## Data Availability

The preprocessed datasets and source code are available at https://github.com/gracygyx/HIGSyn.
