# Supplementary material for: HIG-Syn: a hypergraph and interaction-aware multigranularity network for predicting synergistic drug combinations
Source: Bioinformatics. 2025 Jul 15;41(Suppl 1):i86–95. doi: 10.1093/bioinformatics/btaf215 (PMC12261487; doi:10.1093/bioinformatics/btaf215)
Supplement: btaf215_Supplementary_Data [file btaf215_supplementary_data.zip › btaf215_Supplementary_Data/Zhang.136.Alt_text_BIOINF-2025-0991.docx]

**Figure 1 caption**

**The architecture of HIG-Syn**. The model input consists of drug molecule graphs for two drugs and cell line gene expression data. (A) The initialization module extracts substructure- and molecular-level features for drugs using GIN layers with SAGPool, while MLP processes cell line features. (B) The coarse granularity module employs a hypergraph for global features, and (C) the fine granularity module focuses on substructure interactions using interaction-aware attention to local features. (D) In the prediction module, coarse and fine features are integrated via a highway network, with MLP layers predicting drug combination outcomes.

**Figure 1 alt text**

Schematic diagram composed of four vertically aligned parts labeled A to D, each representing a key module of the HIG-Syn model. The figure uses colored blocks to represent feature matrices, with arrows indicating the flow of information and network modules representing neural operations. Part A shows drug and cell line encoders; part B shows a hypergraph structure for extracting global features; part C illustrates substructure-level interactions; and part D shows final prediction via highway fusion.

**Figure 2 caption**

**The interaction-aware attention module.**

(a) Cross-attention module for computing substructure-substructure and substructure-cell line interactions. (b) Self-attention module for integrating interaction-aware features and generating the final fine-level representations.

**Figure 2 alt text**

Diagram of cross- and self-attention modules, shown sequentially from (a) to (b). Colored blocks represent input features processed via multi-head attention and linear layers. Arrows indicate data flow, and circular plus icons denote residual connections

**Figure 3 caption**

**Impact of component removal on model performance.**

(a) illustrates the impact of module removal on model performance across classification metrics. (b) illustrates the effect of removing components from the loss function on model performance.

**Figure 3 alt text**

Two bar plots show performance differences across evaluation metrics when (a) a model module or (b) a loss component is removed. Each bar represents a variant of the HIG-Syn model, with color-coded groups and annotated performance difference values. Evaluation metrics shown on the x-axis include AUC-ROC, AUC-PR, Accuracy, BACC, F1 score, Precision, Recall, and Kappa. In both plots, the baseline HIG-Syn serves as the reference point (performance difference = 0), and bars show deviations of ablated variants: HIG-Syn-w/o-Fine, HIG-Syn-w/o-Coarse, HIG-Syn-w/o-Highway, and HIG-Syn-w/o-HyperRes in (a); HIG-Syn-w/o-SimLoss, HIG-Syn-w/o-ContraLoss, and HIG-Syn-w/o-SimContraLoss in (b).

**Figure 4 caption**

**Scatter plot of predicted probabilities under different drug input orders**.

(a) The DrugComb dataset, (b) The GDSC2 dataset.

**Figure 4 alt text**

Two side-by-side scatter plots showing the predicted synergy probabilities under different drug input orders. In both panels, the x-axis indicates predictions for Drug A–Drug B–Cell line, and the y-axis indicates predictions for Drug B–Drug A–Cell line. Panel (a) displays results on the DrugComb dataset in blue, and panel (b) shows results on the GDSC2 dataset in orange. Each point represents a drug pair prediction, and the red dashed line (y = x) indicates perfect order invariance. The data in both plots are tightly aligned along the diagonal, reflecting high model consistency, with PCC values of 0.932 and 0.978 respectively.

**Figure 5 caption**

**Key Molecular Structures Identified by GIN.**

The color intensity indicates the importance of each substructure, with red representing higher importance. (a-c) Substructure importance of Vismodegib across GIN layers. (d-f) Substructure importance of Taselisib across GIN layers.

**Figure 5 alt text**

Six molecular diagrams illustrate substructure importance for two drugs, Vismodegib (top row, (a)–(c)) and Taselisib (bottom row, (d)–(f)), each across GIN Layers 1 to 3. Atoms are color-coded based on importance scores, ranging from blue (low) to red (high), as shown in the accompanying color bar. The visualization reveals layer-wise differences in substructure importance across GIN layers.

**Figure 6 caption**

**T-SNE visualization of drug-drug-cell line triplet representations across different HIG-Syn modules on GDSC2 dataset.** (a) Initialization module output; (b) Coarse-granularity module output with hypergraph. (c) Fine-granularity module output with interaction-aware attention; (d) Final representation vectors for prediction.

**Figure 6 alt text**

Four t-SNE scatter plots showing drug–drug–cell line triplet embeddings from different modules of the HIG-Syn model on the GDSC2 dataset. Each point represents a triplet, color-coded by synergy type: red for antagonism and yellow for synergism. (a) shows embeddings from the initialization module; (b) from the coarse granularity module using hypergraph features; (c) from the fine granularity module using interaction-aware attention-based features; and (d) the final integrated embeddings before prediction. The plots reveal how the learned representations become more structured and clustered as the model progresses.

**Figure S1 caption**

**Model performance under different hyperparameters.** The optimal hyperparameters are indicated by a pentagon.

**Figure S1 alt text**

A grouped bar plot showing the AUC performance of the HIG-Syn model under different hyperparameter settings. The plot includes seven hyperparameter categories: Multi-Head Attention Heads, Loss Weight, Dropout Rate, Learning Rate, GIN Embedding Size, Hypergraph Architectures, and MLP Architectures. Each bar represents the AUC score for a specific hyperparameter value, with error bars indicating standard deviation. Optimal settings are marked with a pentagon symbol above the corresponding bar.

**Figure S2 caption**

**T-SNE visualization of drug-drug-cell line triplet representations across different HIG-Syn modules on DrugComb dataset**. (a) Initialization module output; (b) Coarse-granularity module output with hypergraph. (c) Fine-granularity module output with interaction-aware attention; (d) Final representation vectors for prediction.

**Figure S2 alt text**

Four t-SNE scatter plots showing drug–drug–cell line triplet embeddings from different modules of the HIG-Syn model on the DrugComb dataset. Each point represents a triplet, color-coded by synergy type: blue for antagonism and orange for synergism. (a) shows embeddings from the initialization module; (b) from the coarse granularity module using hypergraph features; (c) from the fine granularity module using interaction-aware attention-based features; and (d) the final integrated embeddings before prediction. The plots reveal how the learned representations become more structured and clustered as the model progresses.

**Table 1 caption**

Atomic features of the drug graph.

**Table 1 alt text**

A table of three columns describes the atomic features used in the drug graph. The columns are: the feature name, the dimensionality of each feature, and encoding details. The five atomic features include atom type, degree, number of hydrogens, implicit valence, and aromaticity, with one-hot or binary encodings used to represent them.

**Table 2 caption**

Performance comparison of HIG-Syn and 11 baseline models on the DrugComb dataset. The best traditional machine learning and deep learning methods are marked with an asterisk (*) and underlined, respectively.

**Table 2 alt text**

A comparative table shows the performance of HIG-Syn and 11 baseline models on the DrugComb dataset. Eight evaluation metrics are reported: AUC-ROC, AUC-PR, ACC, BACC, F1 score, Precision, Recall, and Kappa. The table includes both traditional machine learning models (e.g., RF, XGBoost) and deep learning models (e.g., HypergraphSynergy, MatchMaker). Asterisks (*) and underlines indicate the best-performing traditional and deep learning models, respectively.

**Table 3 caption**

Model performance in the leave-out scenario on the DrugComb dataset.

The best traditional machine learning and deep learning methods are marked with an asterisk (*) and underlined, respectively.

**Table 3 alt text**

A comparative table reports the performance of HIG-Syn and 11 baseline models on the DrugComb dataset under two leave-out scenarios: Leave-Drug-Pair-Out and Leave-Cell-Line-Out. For each scenario, five evaluation metrics are presented: AUC-ROC, AUC-PR, Balanced Accuracy (BACC), F1 score, and Recall. The table includes both traditional machine learning models (e.g., RF, XGBoost) and deep learning models (e.g., HypergraphSynergy, MatchMaker). Asterisks (*) and underlines indicate the best-performing traditional and deep learning models, respectively.

**Table 4 caption**

Impact of model variants on DrugComb dataset. The best results are highlighted in bold.

**Table 4 alt text**

A table summarized the performance of HIG-Syn and its model variants on the DrugComb dataset. Eight evaluation metrics are reported: AUC-ROC, AUC-PR, ACC, BACC, F1 score, Precision, Recall, and Kappa. Model variants include versions using GCN, GAT with 1, 2, or 4 heads, and different pooling methods (AddPool, MaxPool, and MeanPool). The best-performing values for each metric are highlighted in bold.

**Table 5 caption**

Predicted outcomes from the eleven compared models on the twelve novel combinations. The symbols ∗, ∗∗, and ∗ ∗ ∗ indicate that the literature validates the synergistic properties of the listed drug pair in (i) a cell line unrelated to the listed tissue or disease, (ii) the tissue or disease associated with the listed cell line, and (iii) the listed cell line, respectively

**Table 5 alt text**

A table lists twelve novel drug combinations from the DrugComb and GDSC2 datasets, along with the associated cell lines and supporting literature references. The table contains four columns: dataset name, drug pair, cell line, and reference. The symbols ‘∗’, ‘∗∗’, and ‘∗∗∗’ next to the references indicate different levels of experimental validation reported in the literature.

**Table S1 caption**

Hyperparameter settings for HIG-Syn. The optimal configurations are highlighted in bold.

**Table S1 alt text**

A table listing the hyperparameter settings explored for the HIG-Syn model. The table has two columns: hyperparameter name and values considered. Hyperparameters include GIN hidden units, MLP hidden units, hypergraph hidden units, number of attention heads, loss function weights, dropout rate, and learning rate. For each parameter, multiple values were tested, and the optimal configuration is highlighted in bold.

**Table S2 caption**

Performance comparison of HIG-Syn and eleven baseline models on the GDSC2 dataset. The best traditional machine learning and deep learning methods are marked with an asterisk (*) and underlined, respectively.

**Table S2 alt text**

A table shows the performance of HIG-Syn and 11 baseline models on the GDSC2 dataset. Eight evaluation metrics are reported: AUC-ROC, AUC-PR, ACC, BACC, F1 score, Precision, Recall, and Kappa. The table includes both traditional machine learning models (e.g., RF, XGBoost) and deep learning models (e.g., DeepDDS-GCN, HypergraphSynergy). Asterisks (*) and underlines indicate the best-performing traditional and deep learning models, respectively.

**Table S3 caption**

Leave-out testing of HIG-Syn and 11 baseline models on the GDSC2 dataset.

The best traditional machine learning and deep learning methods are marked with an asterisk (*) and underlined, respectively.

**Table S3 alt text**

A comparative table reporting the performance of HIG-Syn and 11 baseline models on the GDSC2 dataset under two leave-out scenarios: Leave-Drug-Pair-Out and Leave-Cell-Line-Out. For each scenario, five evaluation metrics are presented: AUC-ROC, AUC-PR, Balanced Accuracy (BACC), F1 score, and Recall. The table includes both traditional machine learning models (e.g., RF, XGBoost) and deep learning models (e.g., DeepDDS-GCN, HypergraphSynergy). Asterisks (*) and underlines indicate the best-performing traditional and deep learning models, respectively.

**Table S4 caption**

Predicted outcomes from the eleven compared models on the twelve novel combinations given in Table 5, with a synergy cutoff score set at 0.6. Positive outcomes are highlighted in bold.

**Table S4 alt text**

A table reports the predicted synergy scores from eleven compared models for twelve novel drug–drug–cell line combinations, corresponding to those listed in Table 5. The columns represent traditional machine learning and deep learning models. A synergy cutoff of 0.6 is applied; predictions exceeding this threshold are highlighted in bold to indicate positive synergy outcomes.

**Table S5 caption**

Validation of inconsistent predictions between the top three baseline models and our model on novel combinations, with synergistic defined as a score above 0.6, and non-synergistic as a score below 0.4. Scores predicted by our model are highlighted in bold.

**Table S5 alt text**

A table summarizes the predicted synergy scores of HIG-Syn with three baseline models: RF, XGBoost, and MatchMaker, on novel drug-drug-cell line combinations from the DrugComb and GDSC2 datasets. Each row presents a combination and the corresponding predicted scores from each model. Synergistic cases are defined by scores above 0.6, and non-synergistic cases by scores below 0.4. Predictions from HIG-Syn are shown in bold to highlight differences from baseline models.
