# Supplementary material for: HIG-Syn: a hypergraph and interaction-aware multigranularity network for predicting synergistic drug combinations
Source: Bioinformatics. 2025 Jul 15;41(Suppl 1):i86–95. doi: 10.1093/bioinformatics/btaf215 (PMC12261487; doi:10.1093/bioinformatics/btaf215)
Supplement: btaf215_Supplementary_Data [file btaf215_supplementary_data.zip › btaf215_Supplementary_Data/Zhang.136.sup.1.pdf]

Supplementary Materials

1. The Components of the HIG-Syn

1.1 Drug Embedding with Graph Isomorphism Network (GIN)

GIN Layer. The molecular graph of each drug is represented as a graph $G_d = (V_d, E_d)$, where V_d is the set of nodes (atoms) and E_d is the set of edges (bonds) connecting the atoms. The node feature matrix $\mathbf{X}_d \in \mathbb{R}^{|V_d| \times F_d}$ is defined, and $F_d = 78$ denotes the dimensionality of node features. The adjacency matrix $\mathbf{A}^{(\mathrm{GIN})} \in \mathbb{R}^{|V_d| \times |V_d|}$ captures the graph structure, with $A_{ij}^{(\mathrm{GIN})} = 1$ indicating an edge between node i and j, and $A_{ij}^{(\mathrm{GIN})} = 0$ otherwise.

At the l-th layer ($l = 1, 2, 3$) of the Graph Isomorphism Network (GIN), the node feature propagation process is defined as:

$$\mathbf{H}^{(l+1)} = \mathrm{GINConv}\left(\mathbf{H}^{(l)}, \mathbf{A}^{(\mathrm{GIN})}\right).$$

For a specific node i, the update is defined as:

$$\boldsymbol{h}_i^{(l+1)} = f^{(l)}\left((1+\epsilon)\cdot \boldsymbol{h}_i^{(l)} + \sum_{j \in \mathcal{N}(i)} \boldsymbol{h}_j^{(l)}\right),$$

where $f^{(l)} = \sigma(\mathbf{W}_2 \cdot \sigma(\mathbf{W}_1 \cdot \boldsymbol{x} + \boldsymbol{b}_1) + \boldsymbol{b}_2)$, with $\mathbf{W}_1 \in \mathbb{R}^{2d \times F_d}$, $\mathbf{W}_2 \in \mathbb{R}^{d \times 2d}$, $\boldsymbol{b}_1 \in \mathbb{R}^{2d}$ and $\boldsymbol{b}_2 \in \mathbb{R}^{d}$. Here, $d = 128$ represents the embedding dimension of the GIN layer, and σ is the ReLU activation function. $\boldsymbol{h}_i^{(l)}$ is the feature vector of node i at layer l, with $\boldsymbol{h}_i^{(0)} = \mathbf{X}_{d,i}^{'}$ being the normalized feature vector for node i at the input layer. $\mathcal{N}(i)$ represents the set of neighbors of node i.

SAGPool for Subgraph-level Representation in Molecular Graphs. At each layer of the GIN network, node-level pooling is performed using SAGPool to assess the importance of each node through a GCN layer. These importance scores are then utilized to compute a subgraph-level representation by aggregating the node features. This process captures both node-level information and the graph's topological structure.

The importance of node i at layer l ($l = 1, 2, 3$) is computed as:

$$\begin{aligned}\boldsymbol{S}^{(l)} &= \mathrm{GCNConv}\left(\mathbf{H}^{(l)}, \mathbf{A}^{(\mathrm{GIN})}\right)\\ &= \sigma\left(\hat{\mathbf{A}}^{(\mathrm{GIN})}\mathbf{H}^{(l)}\mathbf{W}^{(l)} + \boldsymbol{b}^{(l)}\right),\end{aligned}$$

where $\hat{\mathbf{A}}^{(\mathrm{GIN})}$ is the normalized adjacency matrix:

$$\hat{\mathbf{A}}^{(\mathrm{GIN})} = \mathbf{D}^{-\frac{1}{2}}\mathbf{A}^{(\mathrm{GIN})}\mathbf{D}^{-\frac{1}{2}}.$$

Here, $\mathbf{D}$ is the degree matrix, and $\mathbf{W}^{(l)}$, $\boldsymbol{b}^{(l)}$ are learnable parameters. σ are learnable parameters. The node importance scores $\boldsymbol{S}^{(l)}$ are normalized using the softmax function:

$$\hat{\boldsymbol{S}}^{(l)} = \mathrm{softmax}\left(\boldsymbol{S}^{(l)}\right),$$

where $\hat{\boldsymbol{S}}^{(l)}$ represents the normalized importance scores, and each score $\hat{s}_i^{(l)} \in \hat{\boldsymbol{S}}^{(l)}$ corresponds to the importance of node i.

The subgraph embedding $\mathbf{H}_{\text{sub}}^{(l)}$ at layer l is computed as the weighted sum of the node features:

$$\mathbf{H}_{\text{sub}}^{(l)} = \sum_{i \in V_d} \hat{s}_i^{(l)} \cdot \boldsymbol{h}_i^{(l)}.$$

Thus, the subgraph-level embedding $\mathbf{H}_{\text{sub}}^{(l)} = \text{SAGPool}\big(\mathbf{H}^{(l)}, \mathbf{A}^{(\text{GIN})}\big)$ captures the molecular subgraph's features, integrating both node-level information and topological properties.

Jumping Knowledge (JK-Net) for Graph-level Representation in Molecular Graphs. To capture multi-scale features across different layers, we employ the Jumping Knowledge (JK-Net) mechanism, which concatenates node-level outputs from multiple layers. This mechanism allows for effective cross-layer integration of information. After applying JK-Net, we use SAGPool to obtain the final graph-level representation, preserving both local and global graph structures.

The global graph-level representation is computed as:

$$\mathbf{H}_{\text{drug}}^{(\text{GIN})} = \text{JK}\left(\left[\mathbf{H}_1^{(l)}, \mathbf{H}_2^{(l)}, \mathbf{H}_3^{(l)}\right]\right),$$

where $\text{JK}(\cdot)$ denotes the concatenation of node-level outputs across layers. Subsequently, SAGPool is applied to obtain the final graph-level representation:

$$\mathbf{H}_{\text{drug}}^{(\text{GIN})} = \text{SAGPool}\Big(\mathbf{H}_{\text{global}}, \mathbf{A}^{(\text{GIN})}\Big).$$

This process effectively integrates multi-level feature interactions and ensures a comprehensive representation of the molecular graph.

1.2 Cell Line Embedding with Multilayer Perceptron (MLP)

Cell line embeddings were derived through a two-layer MLP. The gene expression data of the cell line is represented as $\mathbf{X}_c \in \mathbb{R}^{|V_c| \times F_c}$, where $F_c = 954$ denotes the number of genes.

The final cell line feature representation is computed as:

$$\mathbf{H}_{\text{cell}}^{(\text{MLP})} = \sigma\left(\mathbf{W}_2 \cdot \sigma(\mathbf{W}_1 \cdot \mathbf{X}_c + \boldsymbol{b}_1) + \boldsymbol{b}_2\right),$$

where $\mathbf{W}_1 \in \mathbb{R}^{2d \times F_c}$, $\mathbf{W}_2 \in \mathbb{R}^{3d \times 2d}$ and $\boldsymbol{b}_1 \in \mathbb{R}^{2d}$, $\boldsymbol{b}_2 \in \mathbb{R}^{3d}$ are learnable parameters. Here, $d = 128$ is consistent with the embedding dimension used in the GIN layer, and σ denotes the ReLU activation function.

1.3 Coarse Granularity Module with Hypergraph

The drug features, denoted as $\mathbf{H}_{\text{drug}}^{(\text{GIN})}$, are extracted via the Graph Isomorphism Network (GIN) component, while the cell line features, $\mathbf{H}_{\text{cell}}^{(\text{GIN})}$, are obtained using a Multi-Layer Perceptron (MLP) component.

The propagation process for layer l $(l = 1, 2, 3)$ is as follows:

$$\begin{aligned}\mathbf{H}^{(l+1)} &= \text{HypergraphConv}\left(\mathbf{H}^{(l)}, \mathbf{A}^{\text{hyper}}\right)\\ &= \sigma\Big(\mathbf{D}^{-\frac{1}{2}}\mathbf{A}^{(\text{hyper})}\mathbf{D}^{-\frac{1}{2}}\mathbf{H}^{(l)}\mathbf{W}^{(l+1)}\Big).\end{aligned}$$

Here, $\mathbf{H}^{(l)} \in \mathbb{R}^{|V| \times d_l}$ is the node feature matrix at layer l, where $|V|$ denotes the total number of nodes (drugs and cell lines). $\mathbf{A}^{\text{hyper}}$ is the adjacency matrix of the hypergraph, D is the degree matrix, and σ

is the ReLU activation function. The feature dimensions evolve as follows: $\mathbf{H}^{(0)} = [\mathbf{H}_{\text{drug}}^{(\text{GIN})}, \mathbf{H}_{\text{cell}}^{(\text{MLP})}] \in \mathbb{R}^{|V|\times 3d}$, $\mathbf{H}^{(1)}$ and $\mathbf{H}^{(2)} \in \mathbb{R}^{|V|\times 6d}$, and $\mathbf{H}^{(3)} \in \mathbb{R}^{|V|\times 3d}$. The weight matrices are $\mathbf{W}^{(1)} \in \mathbb{R}^{3d\times 6d}$, $\mathbf{W}^{(2)} \in \mathbb{R}^{6d\times 6d}$ and $\mathbf{W}^{(3)} \in \mathbb{R}^{6d\times 3d}$.

For the last layer l ($l = 4$), a residual connection is introduced to prevent the vanishing gradient problem and preserve information from earlier layers:

$$\begin{aligned}\mathbf{H}^{(\text{hyper})} &= \text{HypergraphConv}\left(\mathbf{H}^{(3)} + \mathbf{H}^{(0)}, \mathbf{A}^{(hyper)}\right)\\ &= \sigma\left(\mathbf{D}^{-\frac{1}{2}}\mathbf{A}^{(\text{hyper})}\mathbf{D}^{-\frac{1}{2}}\left(\mathbf{H}^{(3)} + \mathbf{H}^{(0)}\right)\mathbf{W}^{(4)}\right),\end{aligned}$$

where $\mathbf{W}^{(4)} \in \mathbb{R}^{3d\times 3d}$ is the learnable weight matrix for this final layer.

1.4 Fine Granularity Module with Interaction-Aware Attention.

Substructure Embedding Vectors. The substructure embedding vector for drug 1 is denoted as:

$$\mathbf{H}_{drug1}^{(\text{sub})} = \left[\mathbf{H}_{sub,drug1}^{(1)}, \mathbf{H}_{sub,drug1}^{(2)}, \mathbf{H}_{sub,drug1}^{(3)}\right].$$

Similarly, the embedding vector for drug 2, $\mathbf{H}_{drug2}^{(\text{sub})}$, is generated in a same way. The embedding vector of cell line is $\mathbf{H}_{\text{cell}}^{(\text{MLP})}$.

Interactions based on Cross-attention Cross-attention is used to compute interactions between drug substructures, as well as between substructures and the cell line.

The cross-attention interaction between drug 1 and drug 2 substructures is computed as follows:

$$\text{Attn}_{d2,d1} = \text{MultiHeadAttn}\left(\mathbf{H}_{d1}^{(\text{sub})}, \mathbf{H}_{d2}^{(\text{sub})}.\mathbf{H}_{d2}^{(\text{sub})}\right) + \mathbf{H}_{d1}^{(\text{sub})}.$$

Similarly, the interaction from drug 2 substructures to drug 1 substructures is:

$$\text{Attn}_{d1,d2} = \text{MultiHeadAttn}\left(\mathbf{H}_{d2}^{(\text{sub})}, \mathbf{H}_{d1}^{(\text{sub})}.\mathbf{H}_{d1}^{(\text{sub})}\right) + \mathbf{H}_{d2}^{(\text{sub})}.$$

For interactions between drug 1 substructures and the cell line:

$$\begin{aligned}\text{Attn}_{\text{cell},d1} &= \text{MultiHeadAttn}\left(\mathbf{H}_{d1}^{(\text{sub})}, \mathbf{H}_{\text{cell}}^{(\text{sub})}.\mathbf{H}_{\text{cell}}^{(\text{sub})}\right) + \mathbf{H}_{d1}^{(\text{sub})},\\ \text{Attn}_{\text{d1,cell}} &= \text{MultiHeadAttn}\left(\mathbf{H}_{\text{cell}}^{(\text{sub})}, \mathbf{H}_{d1}^{(\text{sub})}.\mathbf{H}_{d1}^{(\text{sub})}\right) + \mathbf{H}_{\text{cell}}^{(\text{sub})}.\end{aligned}$$

The interaction between drug 2 substructures and the cell line is calculated as:

$$\begin{aligned}\text{Attn}_{\text{cell},d2} &= \text{MultiHeadAttn}\left(\mathbf{H}_{d2}^{(\text{sub})}, \mathbf{H}_{\text{cell}}^{(\text{sub})}.\mathbf{H}_{\text{cell}}^{(\text{sub})}\right) + \mathbf{H}_{d1}^{(\text{sub})},\\ \text{Attn}_{\text{d2,cell}} &= \text{MultiHeadAttn}\left(\mathbf{H}_{\text{cell}}^{(\text{sub})}, \mathbf{H}_{d2}^{(\text{sub})}.\mathbf{H}_{d2}^{(\text{sub})}\right) + \mathbf{H}_{\text{cell}}^{(\text{sub})}.\end{aligned}$$

Fusion of Interactions via Self-Attention To fuse these interactions, we concatenate the computed attention values:

$$\begin{aligned}&\mathbf{H}_{\text{com}}\\ &= [\text{Attn}_{d2,d1}, \text{Attn}_{d1,d2}, \text{Attn}_{\text{d1,cell}}, \text{Attn}_{\text{cell},d1}, \text{Attn}_{\text{d2,cell}}, \text{Attn}_{\text{cell},d2}],\end{aligned}$$

where $\mathbf{H}_{\text{com}} \in \mathbb{R}^{6\times 3d}$.

The self-attention mechanism is then applied to this combined representation:

$$\mathbf{H}_{\text{inter}}^{(0)} = \text{MultiHeadAttn}\left(\mathbf{H}_{\text{com}}, \mathbf{H}_{\text{com}}, \mathbf{H}_{\text{com}}\right) + \mathbf{H}_{\text{com}}.$$

Next, a fully connected layer is applied:

$$\mathbf{H}_{\text{inter}}^{(1)} = \sigma\left(\mathbf{W}_1 \cdot \mathbf{H}_{\text{inter}}^{(0)} + \boldsymbol{b}_1\right).$$

Followed by a flattening operation and an additional fully connected layer:

$$\mathbf{H}^{(\text{fine})} = \sigma\left(\mathbf{W}_2 \cdot \text{flatten}\left(\mathbf{H}_{\text{inter}}^{(1)}\right) + \mathbf{b}_2\right),$$

where $\mathbf{W}_1 \in \mathbb{R}^{3d\times 3d}$, $\mathbf{W}_2 \in \mathbb{R}^{18d\times 9d}$, $\mathbf{b}_1 \in \mathbb{R}^{3d}$ and $\mathbf{b}_2 \in \mathbb{R}^{9d}$ are trainable parameters. The number of attention heads in the multi-head attention is set to 4.

This interaction-aware fine granularity representation $\mathbf{H}^{(\text{fine})}$ captures the relationships between drug substructures and their interactions with the cell line.

1.5 Highway Network for Fusion of Granularity-Level Features

The Highway Network is employed to fuse the features extracted from the coarse and fine modules. Let $\mathbf{H}_{\text{dual}}^{(0)}$ denote the concatenation of the representations from two parallel modules:

$$\mathbf{H}_{\text{dual}}^{(0)} = \left[\mathbf{H}^{(\text{hyper})}, \mathbf{H}^{(\text{fine})}\right],$$

where $H^{(\text{hyper})}\ in\mathbb{R}^{9d}$ represents the outputs from the coarse module based on the hypergraph, and $H^{(\text{fine})}\ in\mathbb{R}^{9d}$ corresponds to the fine module outputs obtained via interaction-aware attention.

The initial transformation is computed as:

$$\mathbf{H}_{\text{dual}}^{(1)} = \sigma\left(\mathbf{W}_1 \cdot \mathbf{H}_{\text{dual}}^{(0)} + \boldsymbol{b}_1\right),$$

where $\mathbf{W}_1 \in \mathbb{R}^{9d\times 9d}$ and $\boldsymbol{b}_1 \in \mathbb{R}^{9d}$ are learnable parameters, and σ denotes the ReLU function.

The gating mechanism is defined as:

$$G\left(\mathbf{H}_{\text{dual}}^{(1)}\right) = \sigma\left(\mathbf{W}_G \cdot \mathbf{H}_{\text{dual}}^{(0)} + \boldsymbol{b}_G\right),$$

where $\mathbf{W}_G \in \mathbb{R}^{18d\times 18d}$ and $\boldsymbol{b}_G \in \mathbb{R}^{18d}$, with σ being the sigmoid function. The gate G controls how much of the transformed output is retained.

The transformed output is given by:

$$T\left(\mathbf{H}_{\text{dual}}^{(1)}\right) = \sigma\left(\mathbf{W}_T \cdot \mathbf{H}_{\text{dual}}^{(0)} + \boldsymbol{b}_T\right),$$

where $\mathbf{W}_T \in \mathbb{R}^{18d\times 18d}$ is the weight matrix for the transformation and $\boldsymbol{b}_T \in \mathbb{R}^{18d}$. The ReLU function is applied as the activation.

Finally, the highway connection is computed as:

$$\mathbf{H}^{\text{highway}} = \sigma\left(\mathbf{W}^{(l+1)} \cdot \mathbf{H}^{(l)} + \boldsymbol{b}^{(l+1)}\right).$$

This formulation integrates the outputs from both modules, enabling efficient fusion of coarse and fine granularities while preserving essential information.

1.6 Drug Combination Prediction

The synergy prediction is performed using a three-layer fully connected network, formulated as follows:

$$\mathbf{H}^{(l+1)} = \sigma\left(\mathbf{W}^{(l+1)}\dot{\mathbf{H}^{(l)}} + \boldsymbol{b}^{(l+1)}\right),$$

where $l = 1, 2$. The parameters $W^{(1)} \in \mathbb{R}^{18d \times 8d}$, $W^{(2)} \in \mathbb{R}^{18d \times 2d}$ and $\boldsymbol{b}^{(1)} \in \mathbb{R}^{8d}$, $\boldsymbol{b}^{(2)} \in \mathbb{R}^{2d}$ are learnable weights and biases, and σ denotes the ReLU activation function.

For the final layer ($l = 3$):

$$\mathbf{H}^{\text{pre}} = \sigma\left(\mathbf{W}^{(3)}\dot{\mathbf{H}^{(2)}} + \boldsymbol{b}^{(3)}\right),$$

where $W^{(3)} \in \mathbb{R}^{2d \times 1}$, $\boldsymbol{b}^{(3)} \in \mathbb{R}$ and σ represents the sigmoid function, which outputs a probability for the synergy prediction.

1.7 Loss function

The loss function consists of three parts: the classification loss, the reconstruction loss for drug and cell line embeddings and the contrastive loss.

The classification loss.

$$L_{\text{clf}} = \frac{1}{N}\sum_{i=1}^{N}[y_i \log(h_i) + 1 - y_i \log(1 - h_i)],$$

where $h_i \in \mathbf{H}^{\text{pre}}$. N is the number of samples, y_i is the true label of the sample, h_i is the prediction probability of the label.

The reconstruction loss. The reconstruction loss quantifies the difference between the hypergraph-based embedding similarity matrix and the original similarity matrix.

The original drug features are $\mathbf{X}^{\text{FP}} \in \mathbb{R}^{n_d \times 1024}$, which corresponds to 1024-bit ECFP6 fingerprints for n_d drugs. The original cell line features are $\mathbf{X}^{\text{cell}} \in \mathbb{R}^{n_c \times 954}$ which correspond to gene expression of 954 genes with n_c cell lines. The corresponding hypergraph embeddings for drugs and cell lines are denoted as $\mathbf{H}_{\text{cell}}^{(\text{hyper})} \in \mathbb{R}^{n_d \times 3d}$ and $\mathbf{H}_{\text{drug}}^{(\text{hyper})} \in \mathbb{R}^{n_c \times 3d}$ with $d = 128$.

The similarity matrix is calculated as:

$$\begin{aligned}
\text{sim}_{\text{drug}}^{(\text{hyper})} &= \text{Cosine}\left(\mathbf{H}_{\text{drug}}^{(\text{hyper})}\right), \\
\text{sim}_{\text{cell}}^{(\text{hyper})} &= \text{Cosine}\left(\mathbf{H}_{\text{cell}}^{(\text{hyper})}\right), \\
\text{sim}_{\text{drug}} &= \text{Cosine}\left(\mathbf{X}^{\text{FP}}\right), \\
\text{sim}_{\text{cell}} &= \text{Cosine}\left(\mathbf{X}^{\text{cell}}\right),
\end{aligned}$$

where $\text{Cosine}(X) = \frac{XX^T}{\|X\|_2 \|X\|_2^T}$.

And the reconstruction loss is:

$$L_{\text{rec}} =$$
$$\frac{1}{n_d}\sum_{i=1}^{N}\left[\text{sim}_{\text{drug}}\log\text{sim}_{\text{drug}}^{(\text{hyper})} + (1-\text{sim}_{\text{drug}})\log\left(1-\text{sim}_{\text{drug}}^{(\text{hyper})}\right)\right]$$
$$+\frac{1}{n_c}\sum_{i=1}^{N}\left[\text{sim}_{\text{cell}}\log\text{sim}_{\text{cell}}^{(\text{hyper})} + (1-\text{sim}_{\text{cell}})\log\left(1-\text{sim}_{\text{cell}}^{(\text{hyper})}\right)\right].$$

The contrastive loss. Contrastive loss is employed to enhance the separation between positive and negative samples in the hypergraph embedding space. The positive samples $\{h_i{}^{(\text{hyper},(\text{pos})}|i \in \text{positivesamples}\}$ and negative samples $\{h_i{}^{(\text{hyper},(\text{neg})}|i \in \text{negativesamples}\}$ represent the embeddings of the (drug 1, drug 2, cell line) triplets in the hypergraph space. Since the number of positive samples is more than that of negative samples, we randomly take the same number of negative samples as positive samples to calculate the contrastive loss.

The Euclidean distance for positive samples are computed as follows:

$$d_{\text{pos},i} = \|h_i{}^{(\text{hyper},(\text{pos})}\|_2,$$
$$d_{\text{neg},i} = \|h_i{}^{(\text{hyper},(\text{neg})}\|_2,$$

where $i \in \{1, 2, ..., N\}$

The final contrastive loss is defined as:

$$L_{\text{cl}} = \frac{1}{N}\sum_{i=1}^{N}\max\left(0, \lambda + d_{\text{pos},i} - d_{\text{neg},i}\right),$$

where $\lambda = 1.0$ is the margin that ensures a sufficient separation between positive and negative samples in the embedding space.

The final loss. The overall loss function is a weighted sum of the classification loss (L_{clf}), the reconstruction loss (L_{rec}), and the contrastive loss (L_{cl}), where α is a hyperparameter that controls the trade-off between the classification, reconstruction and contrastive components. The final loss is:

$$L = L_{\text{clf}} + \alpha\left(L_{\text{rec}} + L_{\text{cl}}\right).$$

In our model, we set $\alpha = 0.5$.

2. Comparison Models

To evaluate the model's performance, we compare it against four classical machine learning methods: Random Forest (RF), XGBoost, Elastic Net (EN), and Gradient Boosting Machine (GBM), as well as seven state-of-the-art deep learning approaches: Multi-Layer Perceptron (MLP), DeepDDS, DFFNDDS, HypergraphSynergy, Matchmaker, MFSynDCP, MPFFPSDC, and SDDSynergy. The classical methods are implemented using Python's scikit-learn package, while XGBoost is implemented using the Python xgboost package.

- DeepDDS (Wang et al., Briefings in Bioinformatics, 2022): This model employs a graph neural network (GNN) to extract drug features from molecular graphs, while cell line features are generated via a multilayer perceptron (MLP) based on gene expression data. The drug and cell line features are concatenated to predict drug combination synergy. Two variants of the model are proposed:

DeepDDS-GAT, based on a graph attention network (GAT), and DeepDDS-GCN, based on a graph convolutional network (GCN).

- HypergraphSynergy (Liu et al., Bioinformatics, 2022): This model extracts drug and cell line features using a hypergraph, where drugs and cell lines are represented as nodes, and synergistic drug-pair-cell line combinations form hyperedges. By concatenating the extracted features, the model predicts the property of drug combination.

- MatchMaker (Kuru et al., IEEE-ACM TCBB, 2021): This model predicts drug synergy using two Drug-Specific Networks (DSNs) and a Synergy Prediction Network (SPN). Each DSN processes a concatenated vector of drug-specific molecular features and cell line gene expression data. The SPN then takes the output vectors from both DSNs to predict the synergistic effect of the drug combination.

- MFSynDCP (Dong et al., BMC bioinformatics, 2024): The model employs a Graph Attention Network (GAT) to extract drug features from molecular graphs, while genomic features of cell lines are extracted using a Multi-Layer Perceptron (MLP). A graph aggregation-based adaptive attention mechanism identifies key drug substructures and captures interactions between drug pairs. Drug and cell line features are then effectively fused using a gating mechanism to predict drug synergy.

- MPFFPSDC (Bao et al., Methods, 2023): The model employs a Graph Convolutional Network (GCN) to extract drug features from molecular graphs, while cell line features are obtained through a Multi-Layer Perceptron (MLP). A novel multi-head drug-gene attention pooling mechanism integrates cell line features with drug atom-level representations. Furthermore, a multi-feature fusion mechanism combines drug features across multiple dimensions, enhancing the model's ability to predict drug synergy.

- SDDSynergy (Liu et al., Journal of Chemical Information and Modeling, 2024): The model extracts drug substructure features of varying sizes and shapes from molecular graphs using a Substructure Information Passing Network (SIPN) module. Cell line gene expression features are extracted via an MLP module. The synergy prediction of drug combinations is formulated as predicting the effect of drug substructures on cell lines through a drug action module. Within this module, a novel drug-cell line attention mechanism identifies key substructures and their interactions. Finally, the model predicts the effects of drug combinations using a fully connected (FC) layer.

3. Ablation Study

To evaluate the contributions of model components, we conducted ablation experiments using 14 model variants on the DrugComb dataset.

- **HIG-Syn-w/o-Coarse:** A model variant that relies solely on the Fine Granularity Module for predicting synergistic drug combinations.

- **HIG-Syn-w/o-HyperRes:** A model variant without the residual connection in the hypergraph.

- **HIG-Syn-w/o-Fine:** A model variant that relies solely on the Coarse Granularity Module for predicting synergistic drug combinations.

- **HIG-Syn-w/o-Highway:** A model variant where the outputs of the Coarse and Fine Granularity Modules are concatenated directly without the use of the Highway Network during prediction.

- **HIG-Syn-w/o-SimLoss:** A model variant where the loss function excludes the reconstruction loss.
- **HIG-Syn-w/o-ContraLoss:** A model variant where the loss function excludes the contrastive loss.
- **HIG-Syn-w/o-SimContraLoss:** A model variant where the loss function excludes the reconstruction and contrastive loss.
- **HIG-Syn-GAT-k:** A variant where GIN is replaced with a GAT for feature extraction, using k attention heads (where k can be 1, 2, or 4).
- **HIG-Syn-GCN:** A variant where GIN is replaced with a GCN.
- **HIG-Syn-AddPool:** A model variant in which the graph-level aggregator in GIN is replaced from SAGpooling to addition.
- **HIG-Syn-MeanPool:** A model variant in which the graph-level aggregator in GIN is replaced from SAGpooling to mean aggregation.
- **HIG-Syn-MaxPool:** A model variant in which the graph-level aggregator in GIN is replaced from SAGpooling to max pooling.

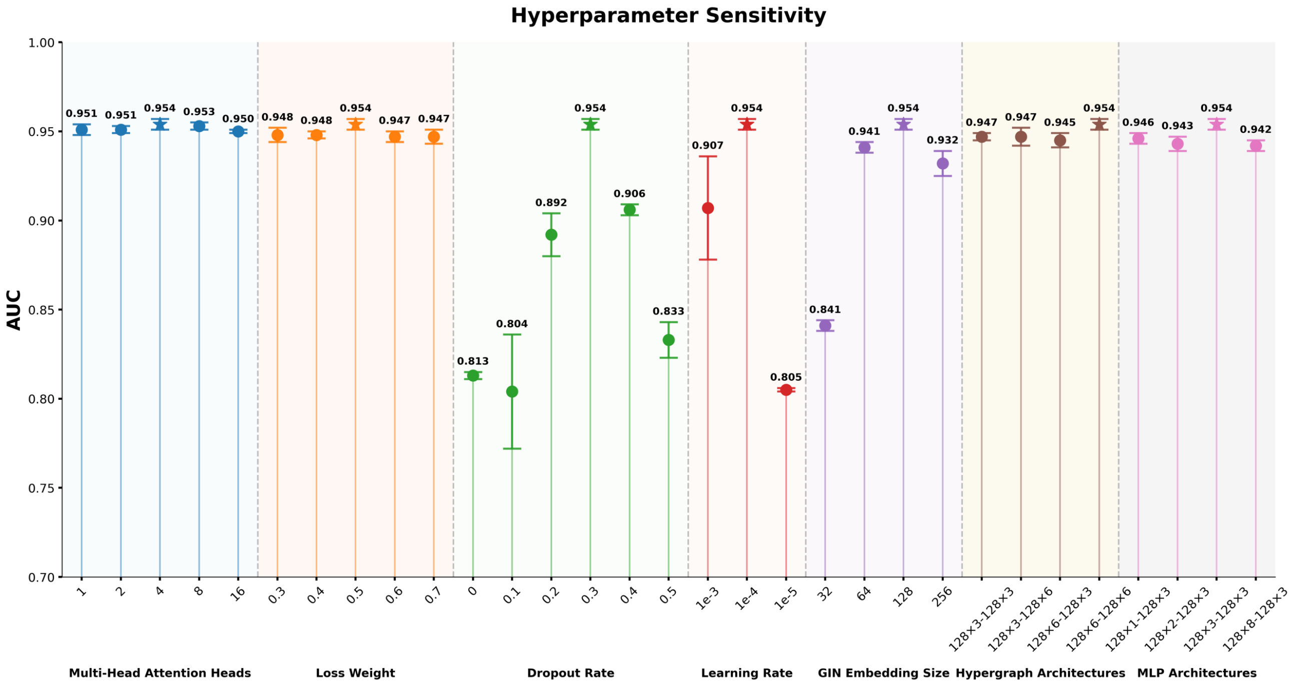

Figure S1. Model performance under different hyperparameters. The optimal hyperparameters are indicated by a pentagon.

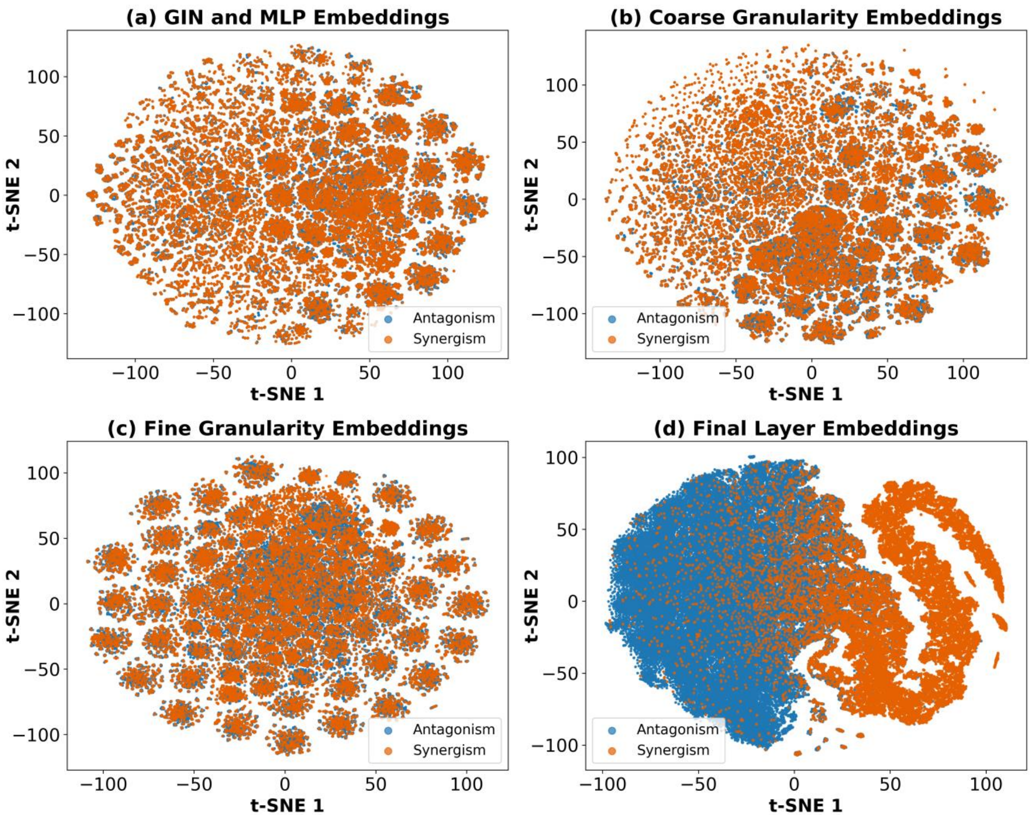

Figure S2. T-SNE visualization of drug-drug-cell line triplet representations across different HIG-Syn modules on DrugComb dataset. (a) Initialization module output; (b) Coarse-granularity module output with hypergraph. (c) Fine-granularity module output with interaction-aware attention; (d) Final representation vectors for prediction.

Table S1. Hyperparameter settings for HIG-Syn. The optimal configurations are highlighted in bold.

Hyperparameter	Values considered
GIN hidden units	[32, 32, 32]; [64, 64, 64]; **[128, 128, 128]**; [256, 256, 256]
MLP hidden units	[64, 384]; **[512, 384]**; [1024. 384]
Hypergraph hidden units	[384, 384, 384, 384]; [384, 768, 384, 384]; **[768, 384, 384, 384]**; [768, 768, 384, 384]
Number of attention heads	1; 2; **4**; 8; 16
Loss function weights	0.3; 0.4; **0.5**; 0.6; 0.7
Dropout rate	0; 0.1; 0.2; **0.3**; 0.4; 0.5
Learning rate	1e-3; **1e-4**; 1e-5

Table S2. Performance comparison of HIG-Syn and eleven baseline models on the GDSC2 dataset. The best traditional machine learning and deep learning methods are marked with an asterisk (*) and underlined, respectively.

Model	AUC-ROC	AUC-PR	ACC	BACC	F1	Precision	Recall	Kappa
HIG-Syn	**0.970**±0.003	**0.970**±0.004	**0.933**±0.006	**0.933**±0.006	**0.932**±0.006	**0.929**±0.010	**0.936**±0.009	**0.865**±0.012
EN	0.710±0.003	0.669±0.005	0.655±0.002	0.655±0.002	0.660±0.001	0.644±0.004	0.676±0.003	0.309±0.005
GBM	0.796±0.002	0.778±0.003	0.723±0.003	0.723±0.003	0.733±0.003	0.700±0.003	0.769±0.008	0.445±0.006
RF	0.862±0.003*	0.854±0.005*	0.782±0.003*	0.783±0.003*	0.783±0.003*	0.774±0.005*	0.793±0.004*	0.565±0.006*
XGBoost	0.862±0.002	0.854±0.003	0.780±0.004	0.780±0.004	0.780±0.003	0.774±0.006	0.787±0.004	0.561±0.008
DeepDDS-GAT	0.935±0.016	0.932±0.016	0.870±0.026	0.870±0.026	0.867±0.029	0.876±0.015	0.858±0.046	0.739±0.052
DeepDDS-GCN	0.953±0.010	0.950±0.011	0.897±0.017	0.897±0.017	0.896±0.017	0.895±0.021	0.896±0.014	0.793±0.034
HypergraphSynergy	0.946±0.004	0.946±0.006	0.891±0.005	0.891±0.005	0.891±0.005	0.884±0.010	0.897±0.007	0.781±0.010
MatchMaker	0.869±0.002	0.865±0.005	0.785±0.002	0.785±0.002	0.783±0.004	0.784±0.013	0.013±0.020	0.571±0.004
MFSynDCP	0.681±0.006	0.642±0.005	0.631±0.005	0.632±0.005	0.636±0.020	0.623±0.012	0.651±0.052	0.052±0.010
MLP	0.840±0.028	0.823±0.038	0.773±0.018	0.773±0.018	0.768±0.020	0.781±0.040	0.760±0.051	0.547±0.036
MPFFPSDC	0.898±0.009	0.895±0.010	0.821±0.010	0.821±0.010	0.817±0.010	0.828±0.016	0.807±0.009	0.642±0.019
SDDSynergy	0.892±0.022	0.882±0.022	0.811±0.031	0.811±0.031	0.810±0.033	0.809±0.042	0.815±0.059	0.622±0.062

Table S3. Leave-out testing of HIG-Syn and 11 baseline models on the GDSC2 dataset. The best traditional machine learning and deep learning methods are marked with an asterisk (*) and underlined, respectively.

Model	Leave-Drug-Pair-Out					Leave-Cell-Line-Out				
	AUC-ROC	AUC-PR	BACC	F1	Recall	AUC-ROC	AUC-PR	BACC	F1	Recall
HIG-Syn	**0.718**± 0.036	**0.702**± 0.046	**0.628**± 0.046	**0.699**± 0.045	**0.871**± 0.059	**0.830**± 0.009	**0.822**± 0.020	**0.740**± 0.010	**0.766**± 0.014	**0.860**± 0.012
EN	0.604± 0.016	0.580± 0.056	0.563± 0.019	0.554± 0.075	0.610± 0.217	0.692± 0.014	0.649± 0.024	0.642± 0.009	0.650± 0.018	0.671± 0.027
GBM	0.632± 0.036	0.597± 0.063	0.589± 0.015	0.562± 0.046	0.547± 0.097	0.765± 0.015	0.744± 0.023	0.699± 0.014	0.714± 0.018	0.760± 0.024
RF	0.688± 0.028*	0.667± 0.061*	0.632± 0.018*	0.617± 0.037*	0.612± 0.074	0.831± 0.010*	0.821± 0.020*	0.751± 0.007*	0.755± 0.007*	0.775± 0.017*
XGBoost	0.676± 0.029	0.662± 0.038	0.619± 0.024	0.614± 0.035	0.618± 0.058*	0.816± 0.010	0.805± 0.023	0.738± 0.010	0.738± 0.015	0.747± 0.024
DeepDDS-GAT	0.718± 0.024	0.700± 0.040	0.645± 0.027	0.649± 0.106	0.669± 0.197	0.780± 0.012	0.771± 0.019	0.708± 0.011	0.713± 0.018	0.732± 0.021
DeepDDS-GCN	0.708± 0.020	0.685± 0.051	0.647± 0.013	0.636± 0.057	0.631± 0.127	0.816± 0.008	0.808± 0.018	0.743± 0.007	0.744± 0.003	0.759± 0.028
HypergraphSynergy	0.718± 0.028	0.692± 0.048	0.633± 0.037	0.701± 0.037	0.873± 0.035	0.768± 0.014	0.764± 0.021	0.677± 0.032	0.725± 0.013	0.860± 0.038
MatchMaker	0.687± 0.057	0.663± 0.038	0.628± 0.038	0.623± 0.046	0.626± 0.102	0.821± 0.007	0.809± 0.021	0.741± 0.008	0.738± 0.018	0.736± 0.024
MFSynDCP	0.669± 0.053	0.637± 0.080	0.600± 0.033	0.528± 0.140	0.496± 0.237	0.682± 0.017	0.639± 0.033	0.614± 0.030	0.579± 0.098	0.552± 0.160
MLP	0.622± 0.025	0.596± 0.061	0.590± 0.021	0.578± 0.031	0.571± 0.040	0.759± 0.009	0.736± 0.021	0.698± 0.006	0.691± 0.020	0.683± 0.040
MPFFPSDC	0.730± 0.035	0.705± 0.060	0.651± 0.031	0.650± 0.039	0.668± 0.114	0.773± 0.007	0.763± 0.021	0.704± 0.007	0.703± 0.016	0.711± 0.038
SDDSynergy	0.709± 0.029	0.696± 0.040	0.646± 0.031	0.630± 0.060	0.625± 0.113	0.802± 0.020	0.791± 0.031	0.724± 0.020	0.712± 0.038	0.692± 0.061

Table S4. Predicted outcomes from the eleven compared models on the twelve novel combinations given in Table ??, with a synergy cutoff score set at 0.6. Positive outcomes are highlighted in bold.

Drug Pair	Cell Line	EN	GBM	RF	XGBoost	DeepDDS -GAT	DeepDDS -GCN	Hypergraph Synergy	MatchMaker	MFSynDCP	MLP	MPFFPSDC	SDDSynergy
1,25-dihydroxy vitamin D3 + Ruxolitinib	T-47D	0.405	0.370	0.423	0.538	0.361	**0.793**	0.146	0.641	0.189	0.000	0.345	0.488
Dactolisib + Crizotinib	T-47D	0.474	0.465	**0.768**	**0.879**	**0.939**	**0.828**	0.577	0.514	0.194	**1.000**	**0.759**	**0.917**
Carboplatin + Ruxolitinib	SK-OV-3	0.327	0.307	0.680	0.310	0.649	**0.850**	**0.829**	0.439	0.068	**1.000**	**0.990**	**0.737**
Crizotinib + Erlotinib	SK-OV-3	0.507	0.487	**0.868**	0.506	**0.930**	0.526	0.607	0.453	0.095	**0.999**	0.615	0.680
Navitoclax + Ruxolitinib	SK-OV-3	0.561	0.503	0.641	0.642	0.585	**0.760**	**0.729**	0.521	0.118	**0.891**	0.451	**0.835**
Vincristine + Crizotinib	HT-29	0.529	0.553	**0.902**	**0.723**	**0.942**	**0.813**	**0.817**	0.640	0.304	**1.000**	0.332	0.561
5-Fluorouracil + AZD1775	HT-29	0.581	0.631	0.760	0.835	**1.000**	0.374	**0.993**	**0.714**	0.365	0.349	**0.734**	0.551
AZD1775 + Doxorubicin	HT-29	**0.704**	**0.841**	**0.895**	**0.989**	**1.000**	**0.986**	**1.000**	**0.771**	0.356	**1.000**	**1.000**	**0.863**
AZD6738 + Paclitaxel	C32	0.638	**0.706**	**0.725**	**0.887**	**1.000**	0.168	**1.000**	**0.849**	0.246	**1.000**	**1.000**	**0.903**
AZD6738 + Etoposide	HT-29	0.636	**0.775**	**0.795**	**0.986**	**1.000**	**1.000**	**1.000**	0.603	0.328	**1.000**	**1.000**	**0.974**
5-Fluorouracil + AZD6738	MHH-ES-1	0.684	**0.730**	**0.780**	**0.922**	**1.000**	**0.920**	**1.000**	0.411	0.640	**0.939**	**1.000**	**0.894**
AZD4320 + AZD5991	MHH-ES-1	0.672	**0.757**	**0.750**	**0.935**	**1.000**	**0.966**	0.322	0.614	0.017	**1.000**	**1.000**	**0.896**

Table S5. Validation of inconsistent predictions between the top three baseline models and our model on novel combinations, with synergistic defined as a score above 0.6, and non-synergistic as a score below 0.4. Scores predicted by our model are highlighted in bold.

Dataset	Drug Pair	Cell Line	HIG-Syn	RF	XGBoost	MatchMaker
DrugComb	1429617-90-2 + Crizotinib	T-47D	**0.000**	0.685	0.810	0.603
	7728-73-6 + Crizotinib	HT-29	**0.000**	0.778	0.661	0.605
GDSC2	AZD1775 + Lapatinib	HT-29	**0.066**	0.685	0.844	0.906
	AZD1775 + Taselisib	MHH-ES-1	**0.022**	0.835	0.982	0.642
	AZD4320 + Dasatinib	HT-29	**0.058**	0.645	0.609	0.788
	AZD4320 + MK-2206	C32	**0.001**	0.780	0.736	0.612
	AZD4320 + SCH772984	C32	**0.154**	0.620	0.764	0.778
	AZD4320 + Trametinib	C32	**0.007**	0.665	0.603	0.833
	AZD6738 + Crizotinib	HT-29	**0.082**	0.620	0.724	0.637
	AZD6738 + Lapatinib	HT-29	**0.001**	0.645	0.703	0.708
	AZD6738 + MK-2206	HT-29	**0.281**	0.745	0.912	0.613
